# Profiling the bacteriome of a diet fed in meal or pelleted form, delivered as dry, wet/dry, or liquid feed and its impact on the fecal and intestinal bacteriome of grow-finisher pigs

**DOI:** 10.1093/jas/skaf461

**Published:** 2026-01-09

**Authors:** James T Cullen, Peadar G Lawlor, Paul Cormican, Gillian E Gardiner

**Affiliations:** Eco-Innovation Research Centre, Department of Science, South East Technological University, County Waterford, X91K0EK, Ireland; Teagasc Pig Development Department, Animal and Grassland Research and Innovation Centre, Moorepark, Fermoy, County Cork, P61C996, Ireland; Teagasc Pig Development Department, Animal and Grassland Research and Innovation Centre, Moorepark, Fermoy, County Cork, P61C996, Ireland; Animal and Bioscience Research Department, Animal and Grassland Research and Innovation Centre, Teagasc Grange, Dunsany, County Meath, C15 PW93, Ireland; Eco-Innovation Research Centre, Department of Science, South East Technological University, County Waterford, X91K0EK, Ireland

**Keywords:** bacteriome, feed, grow-finisher, meal, microbiome, pellets

## Abstract

Research is limited on how feed-associated microbes impact the intestinal bacteriome, growth, and feed efficiency of pigs. The aims of this study were to (1) profile the bacteriome of a meal or pelleted diet, delivered as dry, wet/dry or liquid feed using 16S rRNA gene sequencing; (2) determine its impact on the fecal and intestinal bacteriome of grow-finisher pigs; and (3) investigate if differentially abundant bacterial taxa are correlated with growth parameters of these pigs. The experiment was a 2 x 3 factorial arrangement, with two factors for feed form (meal, pellets) and three factors for feed delivery (dry, liquid, wet/dry). It involved 216 Danavil Duroc × (Large White × Landrace) pigs penned in same-sex pen groups of 6 pigs of similar weight (average ∼33.3 kg). Pen groups were blocked by sex and weight before being randomly assigned to 1 of 6 wheat-barley-soya-based dietary treatments in a completely randomized block design: (1) Dry meal; (2) Dry pellets; (3) Liquid meal; (4) Liquid pellets; (5) Wet/dry meal; (6) Wet/dry pellets. Diets were fed on an *ad-libitum* basis for 64 d. Liquid feed was prepared at a water:feed ratio of 2.5:1 (fresh matter basis). Dry feed was sampled from silos and bags, and liquid feed from mixing tanks and troughs. Bacterial richness was lower in the dry pellets compared to meal (*P* ≤ 0.05). The liquid feed bacteriome was more diverse than that of dry feed (*P* ≤ 0.001). *Weissella* and *Leuconostoc* had higher relative abundance (RA) in residual trough-sampled liquid feed compared to mixing tank and fresh trough-sampled feed. The ileal bacteriome was more diverse (*P* ≤ 0.01) in meal-fed than pellet-fed pigs, with higher RA of *Megasphaera* and *Mitsuokella*, while *Streptococcus* and *Escherichia-Shigella* had greater RA in pellet-fed pigs (*P* ≤ 0.01). *Lactobacillus* was enriched in the intestinal digesta of liquid meal-fed pigs (*P* ≤ 0.05), corresponding with its predominance in this diet. Liquid meal-, liquid pellet-, and wet/dry pellet-fed pigs had the highest average daily gain (*P* < 0.001). Feed conversion efficiency (FCE) was better in dry pellet-fed compared to liquid-fed pigs (*P* < 0.001). *Leuconostoc* (associated with feed fermentation) was most abundant in the feces and ileal digesta of liquid-fed pigs and correlated with poorer FCE (*P* ≤ 0.05). The same *Leuconostoc* found in liquid feed were also detected in the digesta and feces of liquid-fed pigs, implicating feed-derived bacteria as a potential cause of the poorer FCE of liquid-fed pigs.

## Teaser

Lactic acid bacteria associated with feed fermentation, including *Lactobacillus, Weissella*, and *Leuconostoc*, predominated in liquid feed for grow-finisher pigs. Increased abundance of these same lactic acid bacteria in the intestinal tract of pigs fed the liquid feed correlated with poorer feed efficiency, linking these bacteria with the poorer feed efficiency observed in liquid-fed grow-finisher pigs.

## Introduction

Diet composition is known to play a major role in influencing the intestinal microbiome. The impact of the intestinal microbiome on growth and feed efficiency in pigs has been studied extensively (McCormack et al., 2017; Tan et al., 2017; Gardiner et al., 2020; Si et al., 2020). However, there is limited research on the impact of the resident feed microbiome on intestinal microbial communities and subsequent potential impacts on growth and feed efficiency in pigs. Pig diets are often fed in pellet form as opposed to in meal form when dry feeding or wet/dry feeding. Pelleting the diet increases nutrient digestibility and improves feed efficiency (Vukmirović et al., 2017). On the other hand, liquid feed is exclusively prepared using meal feed. A recent study by O’Meara et al. (2020a), from which the samples for this study were obtained, investigated the effect of feeding the same diet in meal or pelleted form when delivered as dry, wet/dry, or liquid feed on the growth and feed efficiency of grow-finisher pigs. It found that feed efficiency was optimal when dry or wet/dry feeding a pelleted diet, while growth was maximized by liquid meal feeding. Since wet/dry pellet feeding achieved comparable growth rates to that of liquid feeding while optimizing feed efficiency, this was recommended as the optimal feeding strategy for grow-finisher pigs. Nonetheless, the on-farm feed delivery choice depends on the requirements of the producer. As reviewed in Cullen et al. (2021), dry and wet/dry feeding results in a higher margin over feed than liquid feeding, largely because of the improvement in feed efficiency achieved. However, maximizing growth in pigs to achieve target slaughter weight as quickly as possible is often the priority on ﬁnisher units (e.g. where facilities are highly stocked). In this case, liquid feeding is as cost-effective as dry and wet/dry feeding, due to the increased growth rate observed with liquid feeding. Furthermore, the ability to reduce the cost of diets by incorporating liquid co-products, when available, also makes liquid feeding an attractive option to some.

To our knowledge, this is the first study to profile the gut bacteriome of grow-finisher pigs provided with the same diet in meal or pelleted form when delivered as dry, wet/dry, or liquid feed. There are potentially beneficial microbiological differences between feed forms and the methods by which they are delivered, which could impact positively on the pig gut microbiome. Although O’Meara et al. (2020b) conducted a culture-based assessment of these diets, the aim of this study was to determine the complete bacterial profile of the dietary treatments using high-throughput 16S rDNA amplicon sequencing. In addition, we investigated whether the dietary treatments modulate the intestinal and fecal bacteriome of the pigs and whether particular bacterial taxa are correlated with improved growth, feed efficiency, and/or carcass quality in these pigs. Spontaneous fermentation in liquid feed has previously been associated with amino acid and gross energy losses from the feed. Therefore, it was hypothesized that the liquid feed bacteriome impacts the feed efficiency of liquid-fed pigs, potentially by altering the pig intestinal bacteriome.

## Materials and Methods

### Ethical approval

The study was conducted in accordance with the legislation for commercial pig production set out in the European Communities (Welfare of Farmed Animals) Regulations 2010 and in Irish legislation (SI no. 311/2010). The care and use of the animals were approved by the Teagasc Animal Ethics Committee (approval no. TAEC 107/2015).

### Study design, animal management, diet preparation, and feeding

Experimental design, animal management, feed preparation, and feeding were previously described by O’Meara et al. (2020a). The experiment was a 2 × 3 factorial arrangement, with two factors for feed form (meal and pellets) and three factors for feed delivery (dry, liquid, and wet/dry feeding). Briefly, the cohort of pigs investigated here was one of the batches from the aforementioned study, comprising 216 pigs [Danavil Duroc × (Large White × Landrace); average starting weight of ∼33.3 kg] formed into 36 same-sex (entire male or female) pen groups of 6 pigs of similar weight (*n* = 6 pen replicates per treatment). Pen groups were blocked by sex and weight before being randomly assigned to one of six dietary treatments in a completely randomized block design: (1) Dry meal diet; (2) Dry pelleted diet; (3) Liquid meal diet; (4) Liquid pelleted diet; (5) Wet/dry meal diet; and (6) Wet/dry pelleted diet. The diets were milled using a hammer mill through a 3 mm screen and were fed on an *ad-libitum* basis for 64 d. During a 2-week adaptation period, 10 d prior to the beginning of the experiment, all pigs were fed their respective dry, liquid, or wet/dry diets in meal form. The ingredient and nutrient composition of the wheat-barley-soya-based experimental diet are given in Supplementary Table S1, with the same diet specification used for all treatments. Treatments 1 and 2 were fed from double-space dry feeders. Treatments 5 and 6 were fed from single-space wet/dry feeders that were fitted with a water nipple in the trough so that pigs could mix the dry feed with water at their preferred water:feed ratio. For the liquid treatments, each pen was equipped with a solenoid valve and a short trough fitted with an electronic sensor. The troughs were located on top of rubber mats to help minimize feed wastage. The liquid feed treatments were prepared in separate mixing tanks at a water:feed ratio of 2.5:1 on a fresh matter basis and were fed using an automated liquid feeding system (HydroMix, Big Dutchman, Vechta, Germany). The liquid feed was delivered to troughs fitted with electronic feed sensors using high-pressure air. Average daily gain (ADG), average daily feed intake (ADFI), and feed conversion efficiency (FCE) were calculated as described by O’Meara et al. (2020a) for the entire experimental period for each treatment group. At slaughter, carcass cold weight, lean meat yield, and kill-out yield were determined as described by O’Meara et al. (2020a).

### Feed, fecal, and intestinal digesta sampling

Feed samples from each of the six treatments were collected on day 27 of the experiment for bacteriome analysis. Dry feed samples were collected from each of the feed silos used to store the dry diets in advance of liquid feeding [meal from silo (*n* = 1); pellets from silo (*n* = 1)], as well as from the feed bags [meal (*n* = 1); pellets (*n* = 1)] from which the dry and wet/dry feeders were filled. Liquid feed samples were taken from the mixing tanks [liquid meal (*n* = 1); liquid pellets (*n* = 1)], while freshly delivered (*n* = 2) and residual (*n* = 2) liquid feed samples from two troughs per liquid feeding treatment were also sampled. Residual feed is defined as uneaten feed that remained in the troughs until just prior to the next feeding. All feed samples were transferred aseptically into 1.5 mL sterile Eppendorf tubes and were immediately snap-frozen in liquid nitrogen and stored at −80 °C until DNA extraction.

Pen groups were given a 2-week adjustment period prior to starting the experiment, where all pigs were fed the experimental diet in meal form via dry, liquid, or wet/dry feeding as per their treatment groups so that they were acclimatized to the feed delivery system and the new accommodation. Baseline fecal samples were collected by O’Meara et al. (2020a) 12 d prior to commencement of the experiment, i.e. 2 d after being introduced to the new feed delivery method. Fecal samples were also collected on day 28 and day 63, the latter being the day before slaughter. At each time point, fecal samples were collected from two pigs selected at random from each of 4 pens per treatment group (*n* = 8 pigs per treatment). On day 64, at an average of ∼101 kg live weight, the pigs were slaughtered by CO_2_ stunning followed by exsanguination (O’Meara et al., 2020a). At slaughter, digesta samples were collected from the terminal ileum (1.5 m proximal to the ileocecal valve) and the blind end of the cecum. The number of digesta samples obtained from both the ileum and cecum for each treatment group were as follows: Dry meal (*n* = 7), dry pellets (*n* = 7), liquid meal (*n* = 8), liquid pellets (*n* = 9), wet/dry meal (*n* = 8), and wet/dry pellets (*n* = 6). Samples of ileal and cecal digesta were aseptically transferred to 1.5 mL Eppendorf tubes and were immediately snap-frozen in liquid nitrogen. Samples were transported to the laboratory on dry ice and were stored at −80°C until DNA extraction.

### DNA extraction, library preparation, and amplicon sequencing

DNA extractions from feed, fecal, and intestinal digesta samples were performed using the QIAamp^®^ Fast DNA Stool Mini kit, following the ‘Isolation of DNA from Stool for Pathogen Detection’ protocol as described by Cullen et al. (2022) with a 20-minute bead-beating step. Bacterial communities were profiled via amplicon sequencing of the V3-V4 hypervariable region of the 16S rRNA gene on the Illumina MiSeq platform, according to the Illumina 16S Metagenomic Sequencing Library Preparation Guide, with some modifications, as described by Cullen et al. (2022). Each PCR reaction contained 25 ng of DNA template, and the reaction volume, components, and PCR conditions were the same as those described by Cullen et al. (2022). The 16S PCR products were quality checked and purified as described previously (Cullen et al., 2022). Final libraries were quantified by qPCR, diluted, denatured, and sequenced using 2 × 300 cycle V2 kits in the Teagasc sequencing facility as described by Fouhy et al. (2015) in accordance with standard Illumina sequencing protocols.

### Bioinformatics and statistical analysis

Demultiplexed paired-end 16S rDNA sequences were imported (in Casava 1.8 demultiplexed paired-end format) into QIIME2 v.2020.8.0 (Bolyen et al., 2019). Sequence quality assessment and initial preprocessing, including primer trimming, filtering, dereplication, chimera removal, and merging of paired-end reads, were performed in QIIME2 as previously described (Cullen et al., 2022). Samples from separate sequencing runs were preprocessed separately in QIIME2. The resultant feature tables and representative sequences were then merged using the ‘qiime feature-table merge’ and ‘qiime feature-table merge-seqs’ commands. Taxonomic assignment was performed on the merged representative sequences using a classifier trained on 16S rRNA gene sequences from the SILVA (version 138) database (Quast et al., 2013). Where possible, species level taxonomic assignment of amplicon sequence variants (ASVs) was performed using BLASTN (version 2.15.0+) against the nucleotide collection of the U.S. National Centre for Biotechnology Information (NCBI).

QIIME artefacts (taxonomy, ASV table, metadata, and phylogenetic tree) were imported into R (version 4.2.1) as a phyloseq (McMurdie and Holmes, 2013) object with the qza_to_phyloseq function in the qiime2r package (Bisanz, 2018). Contaminant ASVs, identified using the ‘prevalence’ method in the decontam package (Davis et al., 2018), were removed prior to further analysis. Further preprocessing included removal of ASVs that were not assigned to the kingdom *Bacteria* and removal of ASVs that phylum-level taxonomy was not assigned to. Finally, the filter_taxa function in phyloseq was used to remove ASVs that were not observed more than 3 times in at least 1% of the samples.

Alpha-diversity (observed ASVs, Pielou’s evenness, and Shannon diversity) and beta-diversity, based on unrarefied filtered sequences, were calculated using the phyloseq package. Differences in alpha-diversity metrics for fecal and intestinal digesta samples were analyzed using a linear mixed-effects model using the lmer function in the lme4 package (Bates et al., 2015), with pen as a random effect. Statistical significance between timepoints, feed forms, delivery methods, and their interactions was tested using the ANOVA function in the car package, followed by pairwise comparisons using Tukey’s Honest Significant Difference test with the emmeans package (Lenth, 2020). Differences in alpha-diversity for feed samples were tested using the same method, except differences between feed forms and sampling locations were tested separately, with the other variable considered a random effect in the model. Alpha-diversity was plotted using the ggpubr package (Kassambara, 2020). Beta-diversity was measured using non-metric multidimensional scaling (NMDS) of Bray-Curtis dissimilarity distances and was plotted using the ggplot2 package (Wickham, 2016). Permutational multivariate analysis of variance (PERMANOVA) with 999 permutations was performed to test for differences between samples using the adonis2 function in the vegan package (Oksanen et al., 2020).

Bacterial relative abundance (RA) data were visualized via heatmaps using the Ampviz2 (Andersen et al., 2018) package. The number of feed samples collected was insufficient to perform differential abundance analysis, and therefore, results presented are numerical only. For fecal and intestinal samples, differentially abundant bacterial genera across feed forms, delivery methods, and timepoints were identified using the linear discriminant analysis (LDA) effect size (LEfSe) method in the microbiomeMarker package, based on normalized RA data (Segata et al., 2011; Cao et al., 2022). A non-parametric Kruskal–Wallis rank-sum test (cut-off: *P ≤* 0.01) was used for the identification of significantly different genera across groups. Linear discriminant analysis scores were used to estimate the effect sizes for differentially abundant genera, with an LDA score (log_10_) of 4.0 used as the cut-off value to avoid spurious results. For fecal and intestinal samples, DESeq2 (Love et al., 2014) was implemented in the microeco package (Liu et al., 2021a) to perform multiple pairwise comparisons between treatment groups to test for differentially abundant bacterial genera. The phyloseq object was converted to a microtable object using the file2meco package (Liu et al., 2022). For each sample type (fecal, ileal, and cecal) and fecal sampling time point, the microtable objects were subset to retain only ASVs that had a mean RA of at least 0.01% in at least 10% of samples. The trans_diff class was used to perform differential abundance testing between treatment groups at the genus level using DESeq2 with default settings.

Pearson correlations between differentially abundant bacterial genera in the fecal and intestinal samples were performed against growth rate, feed intake, feed efficiency, and carcass quality parameters in R using the microeco package. The trans_diff class was used to perform differential abundance testing across treatment groups at the genus level using LEfSe with default settings. A trans_env object containing the growth, feed intake, feed efficiency and carcass quality data was generated and correlated with the genus level differential abundance data using the cal_cor function, using false discovery rate multiple testing correction. The trans_distance class was also utilized to generate a Bray-Curtis distance matrix, and correlations between Bray-Curtis distances and growth, feed intake, feed efficiency, and carcass quality data were tested using the Mantel test. It should be noted that since lower FCE values indicate an improvement in feed efficiency, negative correlations between the RA of a genus and FCE values indicate an improvement in feed efficiency.

Growth parameter (ADG, ADFI, and FCE) data from all pigs in the experiment and carcass data (carcass cold weight, lean meat yield, kill-out yield, carcass ADG, and carcass FCE) from pigs sampled at slaughter were analyzed in the Statistical Analysis Systems (SAS) software package version 9.4 (SAS Institute Inc., Cary, North Carolina, United States) using the linear mixed models procedure (PROC MIXED). Treatment, sex, and their interaction were included in the model as fixed effects, while block was included as a random effect, and average initial pig weight was included as a covariate in the model. Pen was the experimental unit in the case of growth parameters, while pig was the experimental unit in the case of carcass parameters. Results are presented as least squares means ± SEM. The Tukey-Kramer adjustment was used to account for multiple comparisons of means. Differences were considered significant at *P* < 0.05 and as tendencies at 0.05 < *P* < 0.10.

## Results

### Bacterial composition and diversity of dietary treatments according to feed form, delivery method, and sampling location

The number of observed ASVs was lower in the dry pelleted diet compared to the dry meal diet, irrespective of sampling location (Supplementary Figure S1a [see online supplementary material for a color version of this figure]; *P ≤* 0.05). However, Shannon diversity and Pielou’s evenness were unaffected, i.e. the increased richness was driven mainly by lowly abundant ASVs. When compared between sampling locations, irrespective of feed form, the alpha-diversity of the dry feed used for the liquid treatments (silo) and for the dry and wet/dry treatments (bagged) was similar (Supplementary Figure S1b, see online supplementary material for a color version of this figure). However, the number of observed ASVs (*P ≤* 0.001) and Shannon diversity (*P ≤* 0.01) were higher in the liquid feed collected from the mixing tank compared to the dry feed from the silo used to prepare it. The number of observed ASVs further increased between the mixing tank feed and the residual liquid feed sampled from the trough (*P ≤* 0.05). The only differences in Pielou’s evenness were found in the residual trough-sampled feed, where it was lower compared to the mixing tank (*P ≤* 0.05) and fresh trough-sampled feed (*P* ≤ 0.05). Finally, Bray-Curtis beta-diversity analysis showed that the dry feed samples (silo and bagged) clustered away from the liquid feed samples, with liquid feed samples clustering based on sampling location (Supplementary Figure S2, see online supplementary material for a color version of this figure).

**Figure 1.**
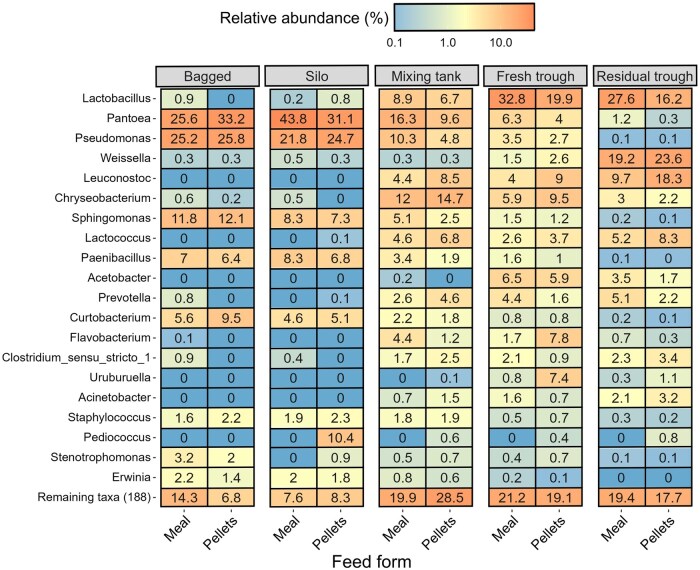
Heatmap displaying the mean relative abundance (%) of the 20 most abundant bacterial genera by feed form (meal or pellets) at each sampling location. Bagged feed (*n* = 2) was used for the dry and wet/dry diets. Feed collected from the silo (*n* = 2) was used to prepare the liquid diets. The mixing tank (*n* = 2), fresh (*n* = 4), and residual trough (*n* = 4) samples were collected for the liquid feed treatments.

Although the number of feed samples collected was insufficient to facilitate differential abundance analysis, the RAs of bacterial genera in the dry meal and pelleted diets were similar (Figure 1). *Pantoea* (25.6% to 43.8% RA), *Pseudomonas* (21.8% to 25.8% RA), and *Sphingomonas* (7.3% to 12.1% RA) were the most abundant genera in the dry feed, irrespective of feed form or sampling location. These were also present in the liquid feed but at a lower RA. Perhaps the most notable difference was the high RA of *Pediococcus* in the pelleted feed collected from the silo (10.4%). It was subsequently only detected in the pelleted liquid feed (at <1% RA).

The bacterial profile of the liquid feed collected from the mixing tanks was similar, irrespective of feed form, with some variation in RA of the predominant genera. For example, in the liquid meal, the RA of *Pantoea* and *Pseudomonas* was approximately twice that of the liquid diet prepared from pellets. In the fresh trough-sampled liquid feed, *Lactobacillus* increased in RA in both the meal (32.8% RA) and pelleted (19.9% RA) diets compared to the mixing tank. Some notable differences between the meal and pelleted diets in the fresh trough-sampled feed included a higher RA of *Flavobacterium* (7.8% vs. 1.7% RA) and *Uruburuella* (7.4% vs. 0.8% RA) in the pelleted compared to the meal diet. A number of genera of lactic acid bacteria (LAB), including *Weissella, Leuconostoc*, and *Lactococcus*, increased in RA in the residual trough-sampled feed compared to the mixing tank and fresh trough-sampled feed. These genera were most abundant in the liquid diet prepared from pelleted feed, while *Lactobacillus* remained more abundant in the residual feed prepared from meal (27.6% vs. 16.2% RA).

### Impact of feed form and delivery method on diversity of pig fecal and intestinal bacteriome

**Figure 2.**
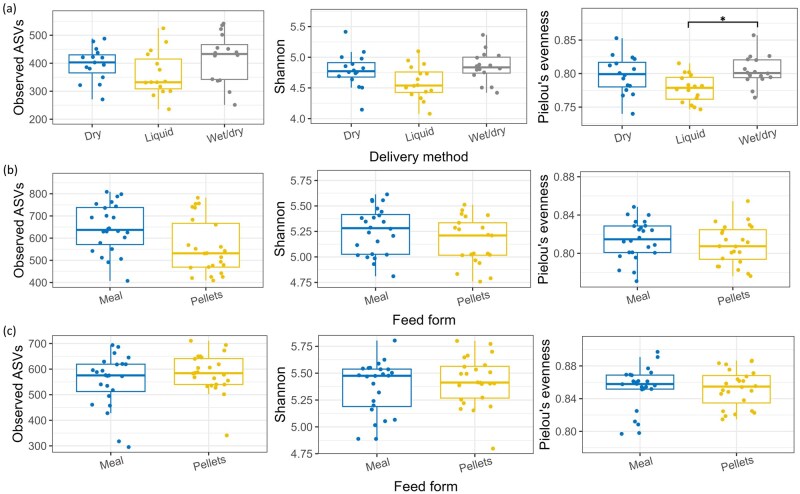
Boxplots displaying alpha-diversity (observed amplicon sequence variants [ASVs], Shannon diversity, and Pielou’s evenness) of the fecal bacteriome of grow-finisher pigs fed dry, liquid, or wet/dry feed in meal or pelleted form. (a) Alpha-diversity by delivery method (dry; *n* = 16, liquid; *n* = 16, or wet/dry; *n* = 16) at baseline (12 d prior to commencement of the experiment, i.e. 2 d after being introduced to the new feed delivery method). All diets were fed in meal form during this period. (b) Alpha-diversity by feed form (meal; *n* = 24, or pellets; *n* = 24, averaged across delivery methods) on day 28. (c) Alpha-diversity by feed form (meal; *n* = 24, or pellets; *n* = 24, averaged across delivery methods) on day 63. *P ≤ 0.05.

During the 2-week adaptation period, 10 d prior to the beginning of the experiment, all pigs were fed their respective dry, liquid, or wet/dry diets in meal form. Therefore, there were no effects of feed form (whether the diet was fed as meal or pellets) on alpha-diversity of the fecal bacteriome during this baseline period. In addition, at baseline, the number of observed ASVs was similar between delivery methods (*P >* 0.05). However, Shannon diversity tended (*P =* 0.06) to be lower, while Pielou’s evenness was lower in the feces of pigs fed liquid feed compared to those fed wet/dry feed (Figure 2a; *P ≤* 0.05). The number of observed ASVs (*P =* 0.06) and Shannon diversity (*P =* 0.09) tended to be lower in the feces of pigs fed pelleted feed compared to those fed meal on day 28 (Figure 2b). When the fecal alpha-diversity of the pigs was examined over time, irrespective of feed form or delivery method, all diversity metrics increased between baseline, day 28, and day 63 of the experiment (Supplementary Figure S3 [see online supplementary material for a color version of this figure]; *P ≤* 0.001), except that the number of observed ASVs was similar between day 28 and day 63.

Both feed form and delivery method had a significant effect on beta-diversity of the fecal bacteriome of pigs fed the experimental diets on day 28 and day 63 (Figure 4a and b). On day 28, feed form had the greatest impact on fecal beta-diversity, explaining 18% of the variation in the bacterial community structure (*R*^2^ = 0.18, *P ≤* 0.001), while feed delivery method explained 7% of the variation (*R*^2^ = 0.07, *P ≤* 0.05). By day 63 of the experiment, both feed form and delivery method impacted the bacterial community structure to a similar extent, both explaining ∼9% of variation (*R*^2^ = 0.09, *P ≤* 0.001 and *R*^2^ = 0.09, *P ≤* 0.1, respectively). When all fecal samples were analyzed together, the sampling time point was found to explain 22% of variance in the bacterial community structure (Supplementary Figure S4a [see online supplementary material for a color version of this figure]; *R*^2^ = 0.22, *P ≤* 0.001), while the feed form and delivery method accounted for 3% (*R*^2^ = 0.03, *P ≤* 0.001) and 2% (*R*^2^ = 0.02, *P ≤* 0.01), respectively.

**Figure 3.**
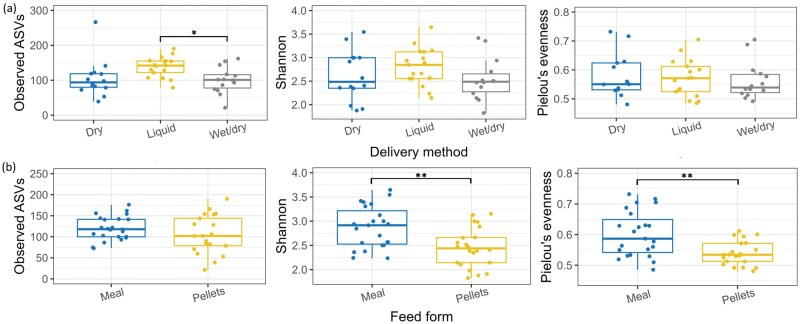
Boxplots displaying alpha-diversity [observed amplicon sequence variants (ASVs), Shannon diversity, and Pielou’s evenness] of the bacteriome in the ileal digesta of grow-finisher pigs fed dry, liquid, or wet/dry feed in meal or pelleted form. (a) Ileal alpha-diversity by delivery method (dry; *n* = 14, liquid; *n* = 17, or wet/dry; *n* = 14), averaged across feed forms. (b) Ileal alpha-diversity by feed form (meal; *n* = 23, or pellets; *n* = 22), averaged across delivery methods. *P ≤ 0.05, **P ≤ 0.01.

**Figure 4.**
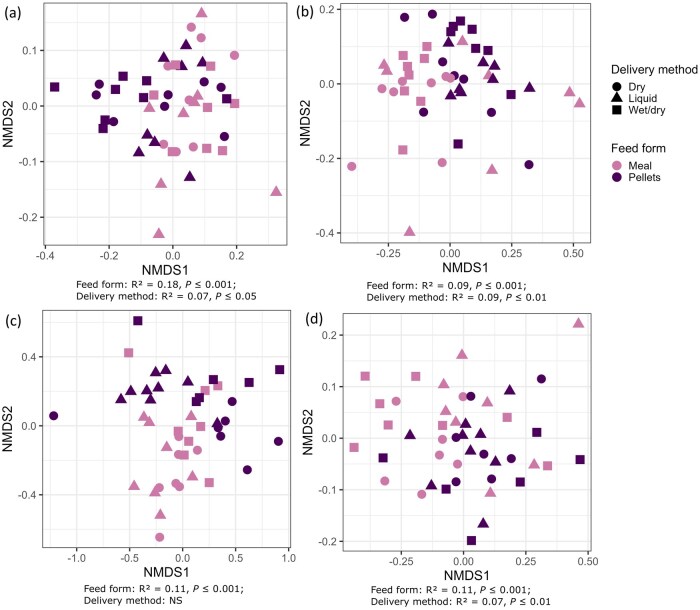
Non-metric multidimensional scaling (NMDS) plots based on Bray-Curtis dissimilarity in the feces (*n* = 48) on (a) day 28 and (b) day 63, and in the (c) ileal (*n* = 45) and (d) cecal (*n* = 45) digesta of grow-finisher pigs. Samples are labelled by feed form (meal or pellets) and delivery method (dry, liquid, or wet/dry feeding) and permutational analysis of variance (PERMANOVA) results for the fecal and intestinal digesta samples are presented below each respective plot.

The number of observed ASVs in the ileal digesta was higher in liquid-fed pigs compared to those fed wet/dry feed (*P ≤* 0.05) and tended to be higher than in those fed dry feed (*P =* 0.08) (Figure 3a). Regarding feed form, both Shannon diversity and Pielou’s evenness were higher in the ileal digesta of pigs fed meal compared to pellets, irrespective of feed delivery method (Figure 3b; *P ≤* 0.01). There were no alpha-diversity differences between feed form or delivery methods in the cecal digesta of the pigs (Supplementary Figure S5 [see online supplementary material for a color version of this figure]; *P >* 0.05). Beta-diversity of the ileal and cecal digesta was influenced by feed form (Figure 4c and d; *R*^2^ = 0.11, *P ≤* 0.001). Delivery method was also found to influence beta-diversity of the cecal digesta (*R*^2^ = 0.07, *P ≤* 0.01). Prior to commencement of the experiment, at baseline, the beta-diversity estimates of the fecal bacteriome were influenced by the feed delivery method, which explained 7% of the variation in the bacterial community structure (Supplementary Figure S4b [see online supplementary material for a color version of this figure]; *R*^2^ = 0.07, *P ≤* 0.01).

### Differential abundance of bacterial genera in intestinal and fecal samples of pigs fed dry, liquid, or wet/dry feed in meal or pelleted form

**Figure 5.**
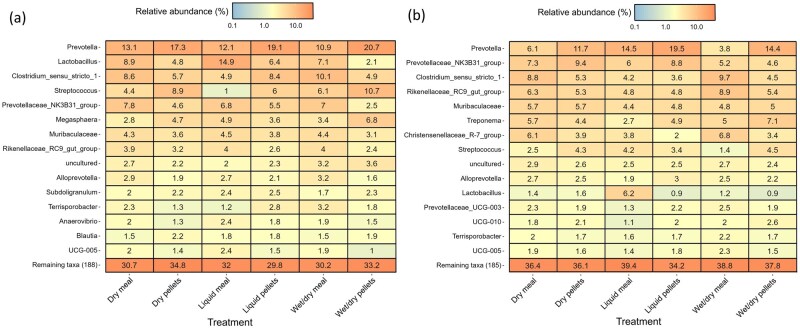
Heatmap displaying the mean relative abundance (%) of the 15 most abundant bacterial genera by treatment group in the feces of grow-finisher pigs fed dry, liquid, or wet/dry feed in meal or pelleted form on (a) day 28 and (b) day 63. In the feces at each time point, *n* = 8 per treatment group.

**Figure 6.**
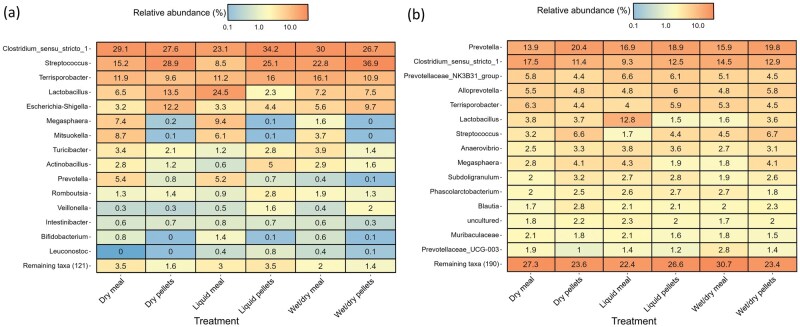
Heatmap displaying the mean relative abundance (%) of the 15 most abundant bacterial genera by treatment group in the (a) ileal and (b) cecal digesta of grow-finisher pigs fed dry, liquid, or wet/dry feed in meal or pelleted form. For each treatment group in the ileal and cecal digesta: Dry meal (*n* = 7), Dry pellets (*n* = 7), Liquid meal (*n* = 8), Liquid pellets (*n* = 9), Wet/dry meal (*n* = 8), Wet/dry pellets (*n* = 6).

**Figure 7.**
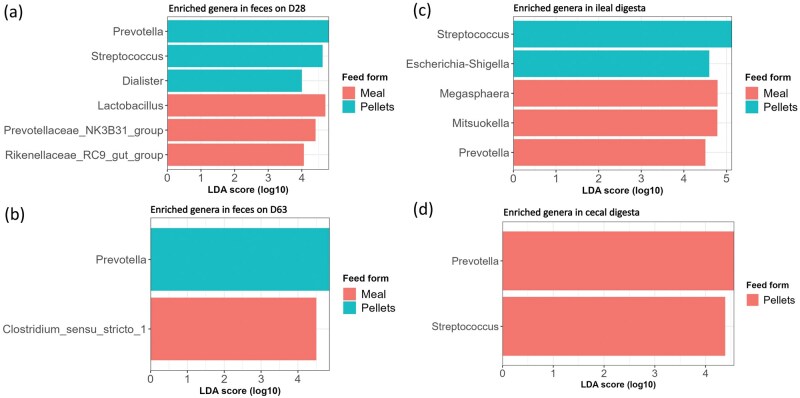
Differentially abundant bacterial genera in the feces on (a) day 28 and (b) day 63, and in the (c) ileal and (d) cecal digesta of grow-finisher pigs fed dry, liquid, or wet/dry feed in meal (feces: *n* = 24; ileal and cecal digesta: *n* = 23) or pelleted (feces: *n* = 24; ileal and cecal digesta: *n* = 22) form. Differential abundances between feed forms were identified by linear discriminant analysis (LDA) with effect size (LefSe). Only genera with an LDA score (log_10_) > 4.0 are shown.

The RA of the 15 most abundant bacterial genera in fecal and intestinal samples of pigs fed the experimental diets are presented by treatment group in Figures 5 and 6, respectively. The data for mean RA of bacterial genera in the fecal, ileal, and cecal samples at each time point by feed form, delivery method, and treatment are available in Supplementary Tables S2–S4. Between treatment group differential abundance analysis results are shown in Supplementary Table S5. Results for the fecal bacteriome at baseline, indicate that *Faecalibacterium* was differentially abundant between delivery methods (*P ≤* 0.01) (Supplementary Figure S6b, see online supplementary material for a color version of this figure), with a mean RA of 3.9% in the feces of liquid-fed pigs compared to 1.4% and 1.8% RA for dry- and wet/dry-fed pigs, respectively (Supplementary Table S2). With respect to feed form, on day 28, *Prevotella, Streptococcus*, and *Dialister* were enriched in the feces of pigs fed pellets (Figure 7a; *P ≤* 0.001), irrespective of delivery method. The RA of *Prevotella* was 19.0% in the feces of pellet-fed pigs, compared to 12.0% in meal-fed pigs (Supplementary Table S3). However, when assessed by delivery method across both feed forms, *Prevotella* was not differentially abundant, with 15.2%, 15.6%, and 15.8% RA found in the feces of dry-, liquid-, and wet/dry-fed pigs, respectively (Supplementary Table S4). In addition to being differentially abundant in the feces of pellet-fed pigs compared to meal-fed pigs (8.5% vs. 3.9% RA), *Streptococcus* was also differentially abundant between feed delivery methods across feed forms on day 28, with the highest RA observed in wet/dry (8.4%) compared to dry (6.6%) and liquid-fed pigs (3.5%) (Supplementary Figure S6b [see online supplementary material for a color version of this figure] and Supplementary Table S4; *P ≤* 0.01). Conversely, *Lactobacillus* was found at a higher RA in meal-fed pigs compared to those fed pellets (10.3% compared to 4.5%) (Figure 7a; *P ≤* 0.001). However, *Lactobacillus* was also differentially abundant between delivery methods across feed forms, with the greatest RA observed in liquid-fed pigs (10.7%) compared to those fed dry (6.9%) and wet/dry feed (4.6%) (Supplementary Figure S6b [see online supplementary material for a color version of this figure] and Supplementary Table S4; *P ≤* 0.01). Between treatments, *Lactobacillus* was more abundant in liquid meal-fed pigs compared to pigs fed dry, liquid, and wet/dry pellets, as well as being enriched in dry meal-fed pigs compared to those fed wet/dry pellets (Supplementary Table S5; *P ≤* 0.05). *Prevotellaceae* NK3B31 group (*P ≤* 0.001) and *Rikenellaceae* RC9 gut group (*P ≤* 0.01) were also differentially abundant between feed forms, with both having greater RA in meal-fed pigs (7.2% and 4.0% RA, respectively), compared to those fed pellets (4.2% and 2.7% RA, respectively) (Figure 7a and Supplementary Table S3).

On day 63, *Clostridium sensu strico 1* was enriched in the feces of pigs fed meal compared to pellets (Figure 7b; *P ≤* 0.01). By treatment, *Clostridium sensu strico 1* was also more abundant in wet/dry meal-fed pigs compared to dry-, liquid-, wet/dry pellet, and liquid meal-fed pigs (Supplementary Table S5; *P ≤* 0.05). As on day 28, *Prevotella* was enriched in pellet-fed pigs compared to those fed meal (*P ≤* 0.001) and *Lactobacillus* was more abundant in the liquid meal-fed pigs compared to all other treatment groups (Supplementary Table S5; *P ≤* 0.05). Several genera including *Lactobacillus, Streptococcus* and *Megasphaera* were differentially abundant in the feces across time points with highest RAs at baseline (Supplementary Figure S6a [see online supplementary material for a color version of this figure]; *P ≤* 0.001). *Treponema, Christensenellenaceae* R-7 group and *Rikenellaceae* RC9 gut group were also differentially abundant across time points, with highest RAs on day 63 (Supplementary Figure S6a [see online supplementary material for a color version of this figure]; *P ≤* 0.001).

*Megasphaera*, *Mitsuokella*, and *Prevotella* were all found at a greater RA in the ileal digesta of meal-fed pigs (6.1%, 6.1%, and 3.6% RA, respectively) compared to those fed pellets (0.1%, 0.07%, and 0.5% RA, respectively) (Figure 7c; *P ≤* 0.001; Supplementary Table S3). *Megasphaera* and *Mitsuokella* had greater RA in the ileal digesta of dry meal-, liquid meal-, and wet/dry meal-fed pigs compared to those fed the pelleted treatments (Supplementary Table S5; *P ≤* 0.05). *Prevotella* followed the same pattern, in that pigs fed meal via dry and liquid feeding had a greater RA of *Prevotella* in their ileal digesta compared to the pelleted treatments; however, this was not the case for wet/dry feeding (Supplementary Table S5; *P ≤* 0.05). Across feed forms, *Streptococcus* (*P ≤* 0.01) and *Escherichia-Shigella* (*P ≤* 0.01) had greater RA in the ileal digesta of pellet-fed pigs (29.5% and 8.3% RA, respectively) compared to meal-fed pigs (15.5% and 4.1%, respectively) (Figure 7c; Supplementary Table S3). By treatment, *Streptococcus* had greater RA in the ileal digesta of dry pellet- and wet/dry pellet-fed pigs, compared to those fed liquid meal (Supplementary Table S5; *P ≤* 0.05). *Escherichia-Shigella* was found at higher RA in the ileal digesta of dry pellet-fed pigs compared to liquid meal-, liquid pellet-, and wet/dry meal-fed pigs (Supplementary Table S5; *P ≤* 0.05).

As with the ileal digesta, no genera in the cecal digesta were differentially abundant according to delivery method. However, when feed forms were compared, both *Prevotella* (*P ≤* 0.01) and *Streptococcus* (*P ≤* 0.01) were enriched in the cecum of pigs fed pelleted compared to meal diets (Figure 7d). *Clostridium sensu strico 1* was found at a higher RA in dry meal-fed pigs (17.5% RA) compared to liquid meal-, liquid pellet-, and dry pellet-fed pigs (9.3% to 14.5% RA) (Supplementary Tables S1 and S4; *P* ≤ 0.05). Additionally, in the cecal digesta, *Lactobacillus* was present at a greater RA in pigs fed liquid meal compared to all other treatments (12.8% vs. 1.5% to 3.8%), except for liquid pellets (Supplementary Table S5; *P* ≤ 0.05).

### Co-occurrence of bacterial ASVs between feed, fecal, and intestinal bacteriomes

The numbers of bacterial ASVs shared between the feed samples and the fecal and intestinal digesta samples are presented in Supplementary Figure S7 (see online supplementary material for a color version of this figure). A total of 60 bacterial ASVs were shared between the feed and the ileal digesta samples, while 152 ASVs were shared between the feed and the cecal digesta samples. A total of 362 ASVs were shared between the feed samples and the fecal samples. An ASV assigned to the genus *Leuconostoc* that was highly abundant in the liquid feed was also detected in the ileal and cecal digesta and fecal samples. Other relevant ASVs that were highly abundant in liquid feed assigned to *Weissella* and *Lactobacillus* were also present in both the intestinal and fecal samples.

### Growth, feed intake, feed efficiency, and carcass quality data

Growth rate, feed intake, and feed efficiency of grow-finisher pigs fed meal or pellets via dry, liquid, or wet/dry feeding are presented in Table 1. Liquid-fed pigs had the highest ADG over the entire experimental period, with values of 1166 and 1160 g/d achieved for the liquid meal- and liquid pellet-fed pigs, respectively (*P ≤* 0.001); however, wet/dry pellet-fed pigs also had similar ADG (1115 g/d; *P ≤* 0.001). The ADFI of liquid meal- and liquid pellet-fed pigs (2713 and 2848 g/d, respectively) was higher than the other treatment groups (*P ≤* 0.001); however, FCE was poorest in the liquid pellet-fed pigs (2.48; *P ≤* 0.001). Feed conversion efficiency was superior in the dry pellet-fed pigs (2.07) compared to the liquid meal- (2.33) and liquid pellet-fed pigs (2.48; *P ≤* 0.001). Although the FCE of the dry pellet-fed pigs was optimal, it was similar to that of the pigs fed dry meal and to those fed meal or pellets via wet/dry feeding (*P ≤* 0.001).

**Table 1.** Average daily gain (ADG), average daily feed intake (ADFI) and feed conversion efficiency (FCE; ADFI/ADG) of grow-finisher pigs fed meal or pellets via dry, liquid or wet/dry feeding over the entire 76-d experimental period, on a pen basis (least squares mean ± SEM; *n* = 6 pens/treatment)

Treatment	ADG, g/d	ADFI, g/d	FCE
**Dry meal**	1033^c^	2280^b^	2.22^b,c^
**Dry pellets**	1072^b,c^	2232^b^	2.07^c^
**Liquid meal**	1166^a^	2713^a^	2.33^a,b^
**Liquid pellets**	1160^a^	2848^a^	2.48^a^
**Wet/dry meal**	1036^b,c^	2340^b^	2.26^b,c^
**Wet/dry pellets**	1115^a,b^	2424^b^	2.17^b,c^
**SEM**	18.7	56.1	0.042
***P*-value**	<0.001	<0.001	<0.001

a,b,c Within each column, values that do not share a common superscript are significantly different (*P <* 0.05).

Carcass characteristics of grow-finisher pigs fed meal or pellets via dry, liquid, or wet/dry feeding are presented in Table 2. Carcass ADG was higher in the liquid-fed pigs, with growth rates of 938 and 958 g/d obtained for liquid meal- and liquid pellet-fed pigs, respectively (Table 2; *P ≤* 0.001). Carcass FCE was optimal in the dry pellet-fed pigs (2.67); however, only the carcass FCE of liquid pellet-fed pigs was poorer (3.07; *P =* 0.02). The cold carcass weight of liquid meal- and liquid pellet-fed pigs was higher than that of dry and wet/dry meal-fed pigs and was similar to that of dry and wet/dry pellet-fed pigs (*P ≤* 0.001). The kill-out yield was similar between all treatment groups, except for dry meal-fed pigs (75.9%; *P =* 0.01), with the highest numerical kill-out yields in the liquid meal- and pellet-fed pigs (78.8% and 78.8%, respectively).

**Table 2.** Carcass characteristics of grow-finisher pigs fed meal or pellets via dry, liquid, or wet/dry feeding at slaughter on an individual pig basis^1^ (least squares mean ± SEM)

Treatment	ADG^2^, g/d	FCE^3^	Cold weight, kg	Kill-out yield, %	Lean meat yield, %
**Dry meal**	840^b^	2.94^a,b^	76.1^b^	75.9^b^	58.3
**Dry pellets**	896^a,b^	2.67^b^	79.8^a,b^	77.9^a,b^	58.0
**Liquid meal**	938^a^	3.00^a,b^	82.4^a^	78.8^a^	56.6
**Liquid pellets**	958^a^	3.07^a^	83.7^a^	78.9^a^	57.2
**Wet/dry meal**	844^b^	2.84^a,b^	76.4^b^	76.6^a,b^	58.3
**Wet/dry pellets**	914^a,b^	2.75^a,b^	80.9^a,b^	77.2^a,b^	57.0
**SEM**	21.1	0.087	1.35	0.69	0.79
***P*-value**	<0.001	0.02	<0.001	0.01	0.53

1 Dry meal and dry pellets: *n =* 7; liquid meal: *n* = 8; liquid pellets: *n* = 9; wet/dry meal: *n* = 8; wet/dry pellets: *n* = 6.

2 Carcass average daily gain.

3 Carcass feed conversion efficiency.

a,b Within each column, values that do not share a common superscript are significantly different (*P <* 0.05).

### Correlation of growth rate, feed intake, and feed efficiency with beta-diversity and differentially abundant bacterial genera in the fecal and intestinal bacteriome

Fecal beta-diversity, as measured by Bray-Curtis distances, of pigs fed the liquid meal treatment was positively correlated with ADG (*r* = 0.46, *P ≤* 0.01), ADFI (*r* = 0.51, *P ≤* 0.01), and FCE (*r* = 0.28, *P ≤* 0.05) on day 28 of the experiment and with ADG (*r* = 0.67, *P* ≤ 0.01) and ADFI (*r* = 0.58, *P* ≤ 0.05) on day 63 (Table 3). These correlations indicate that increased dissimilarity of the fecal bacteriome composition between liquid meal-fed pigs correlates with increased dissimilarity of growth rate, feed intake, and feed efficiency (on day 28) between these pigs. The only other correlation on day 28 was a positive correlation between beta-diversity and FCE in wet/dry pellet-fed pigs (*r* = 0.59, *P ≤* 0.05). On day 63, ADG and ADFI were positively correlated with fecal beta-diversity of pigs fed dry pellets (ADG: *r* = 0.65, *P ≤* 0.01; ADFI: *r* = 0.66, *P ≤* 0.01) and wet/dry meal (ADG: *r* = 0.57, *P ≤* 0.01; ADFI: *r* = 0.54, *P ≤* 0.01). Finally, on day 63, there was a positive correlation between ADG and fecal beta-diversity of liquid pellet-fed pigs (*r* = 0.43, *P ≤* 0.05).

**Table 3.** Mantel test correlation of fecal beta-diversity (Bray-Curtis distances) with average daily gain (ADG), average daily feed intake (ADFI), and feed conversion efficiency (FCE) of grow-finisher pigs fed dry, liquid, or wet/dry feed in meal or pelleted form (*n* = 8 per treatment) on day 28 and day 63

Treatment group	Timepoint	Variable	Correlation coefficient (*r*)	Adjusted *P*-value
**Liquid meal**	Day 28	ADG	0.46	0.006
**Liquid meal**	Day 28	ADFI	0.51	0.006
**Liquid meal**	Day 28	FCE	0.28	0.037
**Wet/dry pellets**	Day 28	FCE	0.59	0.012
**Dry pellets**	Day 63	ADG	0.65	0.003
**Dry pellets**	Day 63	ADFI	0.66	0.006
**Liquid meal**	Day 63	ADG	0.67	0.003
**Liquid meal**	Day 63	ADFI	0.58	0.012
**Liquid pellets**	Day 63	ADG	0.43	0.030
**Wet/dry meal**	Day 63	ADG	0.57	0.003
**Wet/dry meal**	Day 63	ADFI	0.54	0.006

**Figure 8.**
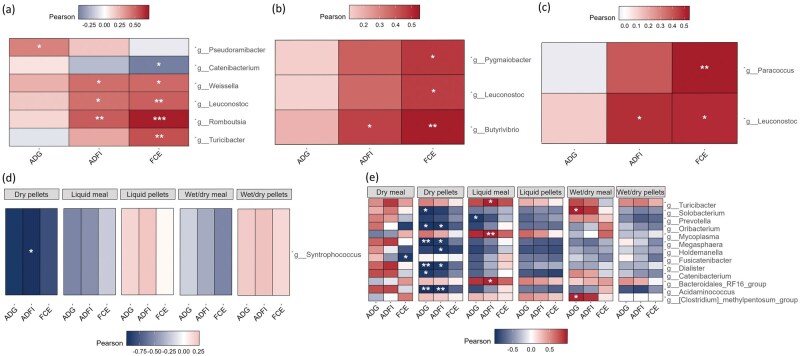
Pearson correlation plots of differentially abundant bacterial genera with average daily gain (ADG), average daily feed intake (ADFI), and feed conversion efficiency (FCE) in the feces and intestinal digesta of pigs fed dry, liquid, or wet/dry feed in meal or pelleted form. Correlations were performed between genera that were differentially abundant across all treatment groups, with ADG, ADFI, and FCE in the feces on (a) day 28; *n* = 48, (b) in the feces on day 63; *n* = 48, and (c) in the ileal digesta; *n* = 45. Correlations were also performed between genera that were differentially abundant in each treatment group with ADG, ADFI, and FCE in the feces on (d) day 28; *n* = 8 per treatment, and (e) day 63; *n* = 8 per treatment. *P ≤ 0.05, **P ≤ 0.01, ***P ≤ 0.001. False discovery rate multiple testing correction was performed. Red indicates a positive correlation, white indicates no correlation and blue indicates a negative correlation. Lower FCE values indicate an improvement in feed efficiency, therefore, negative correlations between the differentially abundant bacterial genera and FCE values indicate improved feed efficiency.

Next, correlation analyses were performed in order to investigate whether the bacterial genera that were enriched in the feces or intestinal digesta between experimental treatment groups were associated with ADG, ADFI, and FCE. It should be noted that since a lower FCE value indicates an improvement in feed efficiency, negative correlations between the RA of a genus and FCE values indicate an improvement in feed efficiency. Conversely, a positive correlation between the RA of a genus and FCE indicates a dis-improvement in feed efficiency. In the feces on day 28, *Pseudoramibacter* was the only differentially abundant genus that was positively correlated with ADG (Figure 8a; *r* = 0.41, *P ≤* 0.05). *Romboutsia* (*r* = 0.53, *P ≤* 0.01)*, Leuconostoc* (*r* = 0.46, *P ≤* 0.01), and *Weissella* (*r* = 0.47, *P ≤* 0.01) in the feces on day 28 were all positively associated with ADFI. The same three genera also had positive correlations with FCE (*r* = 0.73, *P ≤* 0.001; *r* = 0.51, *P ≤* 0.01; *r* = 0.48, *P ≤* 0.01, respectively), in addition to *Turicibacter* (*r* = 0.56, *P ≤* 0.01), meaning that they were associated with poorer feed efficiency.

On day 63, *Butyrivibrio* was associated with poorer feed efficiency, i.e. it had the greatest positive correlation with FCE (Figure 8b, *r* = 0.55, *P ≤* 0.01) and was the only genus positively correlated with ADFI (*r* = 0.46, *P ≤* 0.05). *Leuconostoc* (*r* = 0.47, *P ≤* 0.05) was again associated with poorer feed efficiency in the feces on day 63, in addition to *Pygmaiobacter* (*r* = 0.48, *P ≤* 0.05). In the ileal digesta, *Leuconostoc* was positively correlated with ADFI (*r* = 0.49, *P ≤* 0.01) and associated with poorer feed efficiency (*r* = 0.50, *P ≤* 0.01), while *Paracoccus* in the ileal digesta was also associated with poorer feed efficiency (*r* = 0.54, *P ≤* 0.01). No significant correlations with ADG, ADFI or FCE were found in either the ileal or cecal digesta between individual treatment groups.

There were a number of bacterial genera in the feces that were correlated with production metrics on day 63. Most notably, several genera were negatively correlated with ADG and ADFI in the dry pellet-fed pigs (Figure 8e). These included *Acidaminococcus* (ADG: *r* =* −*0.99, *P ≤* 0.001; ADFI: *r* =* −*0.97, *P ≤* 0.01)*, Dialister* (ADG: *r* =* −*0.99, *P ≤* 0.001; ADFI: *r* =* −*0.94, *P ≤* 0.05), and *Megasphaera* (ADG: *r* =* −*0.99, *P ≤* 0.001; ADFI: *r* =* −*0.92, *P ≤* 0.05). *Turicibacter*, which was associated with poorer feed efficiency on day 28, was positively correlated with ADFI in the liquid meal-fed pigs on day 63, along with *Mycoplasma* (*r* = 0.97, *P ≤* 0.01) and *Bacteroidales RF16 group* (*r* = 0.94, *P ≤* 0.05). *Solobacterium* (*r* = 0.94, *P ≤* 0.05) and *Clostridium methylpentosum* group (*r* = 0.93, *P ≤* 0.05) were both positively associated with ADG in the feces of pigs fed wet/dry meal on day 63. *Catenibacterium*, which was associated with improved feed efficiency in the feces on day 28, was negatively correlated with ADG in dry pellet-fed pigs on day 63 (*r* = 0.93, *P ≤* 0.05), while *Fusicatenibacter* was associated with improved feed efficiency in dry meal-fed pigs (*r* = 0.94, *P ≤* 0.05). Finally, *Prevotella* was found to be negatively correlated with ADG in the feces of liquid meal-fed pigs on day 63 (*r* = 0.93, *P ≤* 0.05).

## Discussion

This study investigated, for the first time, the bacteriome of the same diet in meal or pelleted form delivered as dry, wet/dry, or liquid feed and the impact of feeding these diets on the fecal and intestinal bacteriome of grow-finisher pigs. The objective was to determine the bacterial diversity and composition of the feed itself, profile the fecal and intestinal bacteriome of pigs fed these diets, and investigate whether certain bacterial taxa were associated with improved growth, feed efficiency or carcass quality parameters. Overall, liquid feed had greater bacterial diversity compared with dry feed, with meal supporting a more diverse bacteriome than pellets. Both feed form and delivery method influenced fecal and intestinal bacteriomes, with meal diets and liquid feeding generally supporting higher ileal ­diversity and greater RA of *Lactobacillus*, *Megasphaera*, and *Mitsuokella*, while pelleted diets promoted *Prevotella*, *Streptococcus*, and *Escherichia–Shigella*, particularly in the ileum and cecum. Liquid-fed pigs achieved the highest growth rates, while dry pelleted diets produced the best feed efficiency.

Generally, the bacterial profile of the dry meal and pellets was similar, although *Pediococcus* was present at a higher RA in the one pelleted diet sample collected from the silo. Alpha-diversity analysis showed that the number of observed bacterial ASVs was lower in the pelleted compared to the meal feed. Similarly, the study from which these samples were sourced (O’Meara et al., 2020a) found using culture-based analysis that LAB, *Enterobacteriaceae*, yeast, and mold counts were lower in the pelleted compared to the meal diet (O’Meara et al., 2020a). Although not a measure of decreased abundance of specific taxa, the lower number of observed ASVs found in the pelleted diet in the current study indicates lower species richness. Both the findings here and those of the previous related study are in line with several other studies (Mikkelsen et al., 2004; Canibe et al., 2005; Burns et al., 2015), with the lower microbial load and number of different species in pelleted diets likely resulting from the high temperature and pressure used in the pelleting process. The most notable difference in the feed bacteriome was found in the liquid feed sampled from the troughs compared to the mixing tanks, with several LAB, including *Weissella*, *Leuconostoc*, and *Lactococcus* proliferating in the troughs, indicating the occurrence of spontaneous fermentation. This is a common finding in liquid feed (O’Meara et al., 2020b; Torres-Pitarch et al., 2020a; Cullen et al., 2025).

The dietary treatments had a greater impact on alpha-diversity in the ileum compared to the cecum and feces. The fact that the ileal digesta was more diverse in pigs fed meal compared to pellets was in line with the higher species richness found in the meal diet. Similarly, the finding that liquid-fed pigs had a more diverse ileal bacteriome compared to those fed dry or wet/dry feed corresponded with the higher alpha-diversity observed in liquid feed. The fact that community evenness was lower in the feces of liquid-fed pigs at baseline (fewer bacterial taxa dominated) may have been influenced by the decreased evenness in the residual trough-sampled liquid feed, where LAB, including *Lactobacillus, Weissella*, and *Leuconostoc* predominated.

The growth of pigs from the cohort used for sampling in the current study (*n* = 6 pens of pigs per treatment) was in line with that reported by O’Meara et al. (2020a), where growth data from two batches of grow-finisher pigs fed the same experimental diets (*n* = 12 pens of pigs per treatment) was reported. The fecal beta-diversity of several treatment groups was associated with pig growth performance metrics. For example, fecal beta-diversity of pigs fed the liquid meal diet was positively correlated with ADG, ADFI, and FCE. Optimal FCE was observed in the dry pellet-fed pigs, with a positive correlation between fecal beta-diversity and ADG and ADFI, while beta-diversity was positively correlated with FCE in the wet/dry pellet-fed group. The positive correlation of fecal beta-diversity with measures of growth, feed intake, and feed efficiency here indicates that dissimilarity of the microbial community composition between samples was related to the dissimilarity between the respective performance metrics of the pigs in that group, i.e. indicating a relationship between the two. However, more specific associations between performance metrics and individual bacterial genera will be discussed below.

Overall, the differential abundance of genera was influenced more by feed form than by delivery method. The enrichment of *Streptococcus* and *Escherichia-Shigella* in the ileum of pellet-fed pigs is interesting, as both genera contain species that are potentially pathogenic to pigs. This finding is in line with previous studies where meal feeding has been shown to facilitate lactic acid production in the stomach and small intestine due to increased viscosity and therefore a slower digesta passage rate, aiding acidification of the gastrointestinal contents, thereby promoting pathogen exclusion (Mikkelsen et al., 2004; Canibe et al., 2005; Vukmirović et al., 2017). Although there are limitations to using short-read amplicon data for species-level classification (Martínez-Porchas et al., 2016), the most abundant *Streptococcus* ASV in the ileal and cecal digesta was tentatively identified as *Streptococcus gallolyticus*. Quan et al. (2019) identified *S. gallolyticus* as a potential candidate for improving feed efficiency in pigs. However, Sitthicharoenchai et al. (2022) recently reported it as an emerging pathogen in pigs, responsible for ∼8.6% of bacterial valvular endocarditis cases. The most abundant *Escherichia-Shigella* ASV detected in the ileal and cecal digesta was classified as *Escherichia coli*; however, it is difficult to deduce whether the higher abundance of *E. coli* in pellet-fed pigs is of concern without serological identification, as *E. coli* that inhabit the gastrointestinal tract can be commensal or potentially pathogenic (Abraham et al., 2012).

The enrichment of *Megasphaera* and *Mitsuokella*, two lactate-utilizing bacteria, in the ileal digesta of meal-fed pigs may have been facilitated by the aforementioned increase in lactate production in the small intestine when meal is fed to pigs. *Megasphaera* has previously been positively correlated with carcass weight and butyrate concentration in the cecum of grow-finisher pigs (Torres-Pitarch et al., 2020b). *Mitsuokella* has previously been found at a higher RA in the ileum of more feed efficient pigs, while lower fecal RA was associated with improved feed efficiency (McCormack et al., 2019). *Lactobacillus* was also consistently found at higher RA in the cecal digesta and feces of meal-fed pigs, particularly so in those fed meal via liquid feeding. This is in line with the high RA of *Lactobacillus* observed in the liquid feed collected from the troughs i.e. 32.8% in the fresh liquid meal, compared to <1% in the dry meal or pellets. *Lactobacillus, Megasphaera*, and *Mitsuokella* have been shown to be enriched in the stomach of pigs fed a coarse, non-pelleted diet (Mikkelsen et al., 2007). *Lactobacillus* is often used as a probiotic (Dowarah et al., 2017), and as reviewed by Gardiner et al. (2020), it is consistently more abundant in the cecal and fecal bacteriome of highly feed efficient pigs. It should be noted, however, that several LAB, including some *Lactobacillus* species, are associated with increased bile salt hydrolase activity in the intestine (Geng and Lin, 2016). Overgrowth of these bacteria can lead to poorer lipid metabolism and energy harvest by pigs and this could be a potential driver of the poorer feed efficiency of liquid-fed pigs (He et al., 2017). *Clostridium sensu stricto 1*, another genus enriched in the cecal digesta and feces after feeding meal, compared to pellets is also reportedly more abundant in the cecum and feces of more feed efficient pigs (McCormack et al., 2017; Quan et al., 2018; He et al., 2019), although feeding pellets produced the best feed efficiency in the current study. Additionally, *Clostridium methylpentosum* group, which has been associated with fiber degradation (Liu et al., 2021b), was positively correlated with ADG in the feces of wet/dry meal-fed pigs in the present study.

*Prevotella* and *Streptococcus* were also consistently found at higher RA within the fecal and cecal bacteriome of pellet-fed pigs, which were generally more feed-efficient. Reports on the association between *Streptococcus* and feed efficiency are conflicting (McCormack et al., 2017; Yang et al., 2017; Quan et al., 2018; McCormack et al., 2019). However, in this study, no correlation was found between *Streptococcus* and growth, feed efficiency, or carcass quality. *Prevotella* in the feces was found to be negatively correlated with ADG of pigs fed liquid meal, despite this genus being known to ferment plant-derived polysaccharides in the large intestine, thereby enhancing energy harvest (Amat et al., 2020). Previous studies have associated a *Prevotella*-dominant enterotype with increased feed intake (Yang et al., 2018) and body weight (Mach et al., 2015). However, several studies have also associated *Prevotella* with poorer feed efficiency in pigs (Tan et al., 2017; Yang et al., 2017; Quan et al., 2020). *Dialister* was also more abundant in the feces of pellet-fed pigs and was found to be negatively correlated with ADG and ADFI in dry pellet-fed pigs, which had the best feed efficiency of all treatment groups. Despite the negative association between *Dialister* and ADG and ADFI, the ratio of feed intake to growth rate is what determines feed efficiency. Therefore, although it was not statistically significant in the current study, *Dialister* may play a role in improving feed efficiency. This is supported by previous studies where it was found to be more abundant in the ileum (McCormack et al., 2019) and cecum (Quan et al., 2018) of more feed efficient pigs.

Despite the higher growth rate and feed intake of liquid-fed pigs in this study, poorer feed efficiency was observed in this cohort. This was likely due, at least in part, to increased feed wastage, as previously reported (Russell et al., 1996; Han et al., 2006; L’Anson et al., 2012) and as observed in the study from which the samples for the current study were obtained (O’Meara et al., 2020a). Additionally, losses of energy and amino acids due to spontaneous fermentation in liquid feed may also contribute to poorer feed efficiency in liquid-fed pigs (Canibe and Jensen, 2003; O’Meara et al., 2020a, 2020b, 2020c; Torres-Pitarch et al., 2020a, 2020b; Cullen et al., 2025). Moreover, some bacterial genera that were enriched in the ileal digesta and feces of liquid-fed pigs were associated with poorer feed efficiency in the current study. For example, *Turicibacter* in the feces on day 28 was associated with poorer feed efficiency across all treatments. Decreased RA of *Turicibacter* was associated with increased ADFI in the liquid meal-fed pigs, which may explain the poorer feed efficiency i.e., as a result of increased feed intake. In agreement with the current study, *Turicibacter* has been reported as more abundant in more feed efficient pigs (McCormack et al., 2019) and has been positively correlated with body weight (Wang et al., 2019).

*Leuconostoc*, another genus associated with poorer feed efficiency, was found at greatest RA in the ileal digesta of liquid pellet-fed pigs and was absent in pigs fed dry meal or pellets. The association with poorer feed efficiency may be explained by the higher feed intake associated with increased *Leuconostoc* abundance without a corresponding increase in ADG. *Leuconostoc* has previously been found to be more abundant in the small intestine and colon of pigs fed non-fermented compared to fermented liquid feed (Bunte et al., 2020) (the liquid diet in the current study was non-fermented). To our knowledge, the only correlation between *Leuconostoc* and pig growth metrics to date was reported by Torres-Pitarch et al. (2020), where increased ileal RA of *Leuconostoc mesenteroides* in pigs fed liquid feed was negatively correlated with carcass weight. This finding supports the association of *Leuconostoc* with the poorer feed efficiency observed in this study, as reduced weight combined with increased ADFI will result in poorer feed efficiency. Another interesting finding was that two of the same *Leuconostoc* ASVs that were highly abundant in the liquid feed were also present in the fecal and intestinal digesta of liquid-fed pigs. Therefore, it may be that a microbially-driven impact on feed efficiency is mediated by microbial activity in the liquid feed itself leading to decreased feed nutritional quality, or that the impact is mediated by an altered intestinal bacteriome in liquid-fed pigs. While correlations between *Lactobacillus* and *Weissella* abundance and feed efficiency were not found in this study, ASVs of both genera that were highly abundant in liquid feed were also present in the fecal and intestinal bacteriome. A key question related to the ability of feed-associated microbes to influence feed efficiency is whether they can colonize the intestinal tract of pigs and thus exhibit an effect or if they are only transient. While in this study, we only sampled the luminal digesta, it would be useful to explore the relationship between the feed bacteriome and the mucosa-associated bacteriome, which would be more indicative of intestinal colonization.

## Conclusion

Several LAB including *Weissella, Leuconostoc*, and *Lactococcus* were more abundant in the residual-trough sampled liquid feed, with the greatest RA in the liquid meal diet. Pigs fed the liquid meal diet al.o had a greater RA of *Lactobacillus* in their cecal digesta and feces; however, this was not associated with growth or feed efficiency. Bacterial richness was higher in the meal diet, potentially explaining why the bacteriome of the ileal digesta of meal-fed pigs was more diverse compared to that of pellet-fed pigs. *Leuconostoc*, associated with spontaneous fermentation in liquid feed, was enriched in the ileal digesta and feces of liquid-fed pigs and was correlated with poorer feed efficiency in these animals. The current study also confirms previous findings that liquid feeding meal and wet/dry feeding pellets to grow-finisher pigs produce comparable carcass gain, feed efficiency, and kill-out yield. However, liquid feeding meal optimizes carcass gain, while feeding wet/dry pellets optimizes feed efficiency. This study associates the poorer feed efficiency of liquid-fed pigs with increased ileal and fecal abundance of *Leuconostoc*, with liquid feed being the likely source of this bacterium. Therefore, strategies to reduce spontaneous fermentation in liquid feed may help to improve the feed efficiency of liquid-fed pigs.

## Supplementary Material

skaf461_Supplementary_Data

## Abbreviations:

ADFI: average daily feed intake
ADG: average daily gain
ASVs: amplicon sequence variants
FCE: feed conversion efficiency
LAB: lactic acid bacteria
LDA: linear discriminant analysis
LEfSe: linear discriminant analysis effect size
NCBI: National Centre for Biotechnology Information
NMDS: non-metric multidimensional scaling
PERMANOVA: permutational multivariate analysis of variance
RA: relative abundance

## References

[skaf461-B1] Abraham S. , Gordon D. M., Chin J., Brouwers H. J. M., Njuguna P., Groves M. D., Zhang R., Chapman T. A. 2012. Molecular characterization of commensal *Escherichia coli* adapted to different compartments of the porcine gastrointestinal tract. Appl. Environ. Microbiol. 78(19):6799–6803. doi:10.1128/AEM.01688-12 22798360 PMC3457480

[skaf461-B2] Amat S. , Lantz H., Munyaka P. M., Willing B. P. 2020. *Prevotella* in pigs: the positive and negative associations with production and health. Microorganisms. 8(10):1584. doi:10.3390/microorganisms8101584 33066697 PMC7602465

[skaf461-B3] Andersen K. S. , Kirkegaard R. H., Karst S. M., Albertsen M. 2018. ampvis2: an R package to analyse and visualise 16S rRNA amplicon data. *bioRxiv*. doi:10.1101/299537 (15 December 2025, date last accessed).

[skaf461-B4] Bates D. , Mächler M., Bolker B. M., Walker S. C. 2015. Fitting linear mixed-effects models using lme4. J. Stat. Softw. 67(1):48. doi:10.18637/jss.v067.i01

[skaf461-B5] Bisanz J. E. 2018. qiime2R: Importing QIIME2 artifacts and associated data into R sessions. https://github.com/jbisanz/qiime2R (15 December 2025, date last accessed).

[skaf461-B6] Bolyen E. , Rideout J. R., Dillon M. R., Bokulich N. A., Abnet C. C., Al-Ghalith G. A., Alexander H., Alm E. J., Arumugam M., Asnicar F. et al. 2019. Reproducible, interactive, scalable and extensible microbiome data science using QIIME 2. Nat. Biotechnol. 37(8):852–857. doi:10.1038/s41587-019-0209-9 31341288 PMC7015180

[skaf461-B7] Bunte S. , Grone R., Keller B., Keller C., Galvez E., Strowig T., Kamphues J., Hankel J. 2020. Intestinal microbiota of fattening pigs offered non-fermented and fermented liquid feed with and without the supplementation of non-fermented coarse cereals. Microorganisms. 8(5):638. doi:10.3390/microorganisms8050638 32349407 PMC7284762

[skaf461-B8] Burns A. M. , Lawlor P. G., Gardiner G. E., McCabe E. M., Walsh D., Mohammed M., Grant J., Duffy G. 2015. *Salmonella* occurrence and *enterobacteriaceae* counts in pig feed ingredients and compound feed from feed mills in Ireland. Prev. Vet. Med. 121(3–4):231–239. doi:10.1016/j.prevetmed.2015.07.002 26211839

[skaf461-B9] Canibe N. , Højberg O., Højsgaard S., Jensen B. B. 2005. Feed physical form and formic acid addition to the feed affect the gastrointestinal ecology and growth performance of growing pigs. J. Anim. Sci. 83(6):1287–1302. doi:10.2527/2005.8361287x 15890806

[skaf461-B10] Canibe N. , Jensen B. B. 2003. Fermented and nonfermented liquid feed to growing pigs: effect on aspects of gastrointestinal ecology and growth performance. J. Anim. Sci. 81(8):2019–2031. doi:10.2527/2003.8182019x 12926784

[skaf461-B11] Cao Y. , Dong Q., Wang D., Zhang P., Liu Y., Niu C. 2022. microbiomeMarker: an R/bioconductor package for microbiome marker identification and visualization. Bioinformatics. 38(16):4027–4029. doi:10.1093/bioinformatics/btac438 35771644

[skaf461-B12] Cullen J. T. , Lawlor P. G., Cormican P., Crispie F., Gardiner G. E. 2022. Optimisation of a bead-beating procedure for simultaneous extraction of bacterial and fungal DNA from pig faeces and liquid feed for 16S and ITS2 rDNA amplicon sequencing. Anim. Open Space. 1:100012. doi:10.1016/j.anopes.2022.100012

[skaf461-B13] Cullen J. T. , Lawlor P. G., Cormican P., Gardiner G. E. 2021. Microbial quality of liquid feed for pigs and its impact on the porcine gut microbiome. Animals. 11(10):2983. doi:10.3390/ani11102983 34680002 PMC8532943

[skaf461-B14] Cullen J. T. , Lawlor P. G., Cormican P., Crispie F., Slattery H., Gardiner G. E. 2025. Bacteriome and mycobiome profiling of liquid feed for finisher pigs on commercial pig farms. Sci. Rep. 15(1):24718. doi:10.1038/s41598-025-05928-8 40634391 PMC12241619

[skaf461-B15] Davis N. M. , Proctor D. M., Holmes S. P., Relman D. A., Callahan B. J. 2018. Simple statistical identification and removal of ­contaminant sequences in marker-gene and metagenomics data. Microbiome. 6(1):226. doi:10.1101/221499 30558668 PMC6298009

[skaf461-B16] Dowarah R. , Verma A. K., Agarwal N. 2017. The use of *lactobacillus* as an alternative of antibiotic growth promoters in pigs: a review. Anim. Nutr. 3(1):1–6. doi:10.1016/j.aninu.2016.11.002 29767055 PMC5941084

[skaf461-B17] Fouhy F. , Deane J., Rea M. C., O’Sullivan Ó., Ross R. P., O’Callaghan G., Plant B. J., Stanton C. 2015. The effects of freezing on faecal microbiota as determined using MiSeq sequencing and culture-based investigations. PLoS One. 10(3):e0119355. doi:10.1371/journal.pone.0119355 25748176 PMC4352061

[skaf461-B18] Gardiner G. E. , Metzler-Zebeli B. U., Lawlor P. G. 2020. Impact of intestinal microbiota on growth and feed efficiency in pigs: a review. Microorganisms. 8(12):1886. doi:10.3390/microorganisms8121886 33260665 PMC7761281

[skaf461-B19] Geng W. , Lin J. 2016. Bacterial bile salt hydrolase: an intestinal microbiome target for enhanced animal health. Anim. Health. Res. Rev. 17(2):148–158. doi:10.1017/S1466252316000153 28155801

[skaf461-B20] Han Y. K. , Thacker P. A., Yang J. S. 2006. Effects of the duration of liquid feeding on performance and nutrient digestibility in weaned pigs. Asian-Australas. J. Anim. Sci. 19:396–401. doi:10.5713/ajas.2006.396

[skaf461-B21] He B. , Li T., Wang W., Gao H., Bai Y., Zhang S., Zang J., Li D., Wang J. 2019. Metabolic characteristics and nutrient utilization in high-feed-efficiency pigs selected using different feed conversion ratio models. Sci. China. Life. Sci. 62(7):959–970. doi:10.1007/s11427-018-9372-6 30417245

[skaf461-B22] He Y. , Mao C., Wen H., Chen Z., Lai T., Li L., Lu W., Wu H. 2017. Influence of *ad libitum* feeding of piglets with *Bacillus subtilis* fermented liquid feed on gut flora, luminal contents and health. Sci. Rep. 7:44553. doi:10.1038/srep44553 28291252 PMC5349548

[skaf461-B23] Kassambara A. 2020. ggpubr: ‘ggplot2’ Based Publication Ready Plots. R package version 0.6.0. https://cran.r-project.org/package=ggpubr (15 December 2025, date last accessed).

[skaf461-B24] L’Anson K. A. , Choct M., Brooks P. H. 2012. The influence of particle size and processing method for wheat-based diets, offered in dry or liquid form, on growth performance and diet digestibility in male weaner pigs. Anim. Prod. Sci. 52:899–904. doi:10.1071/AN12082

[skaf461-B25] Lenth R. V. 2020. emmeans: Estimated Marginal Means, aka Least-Squares Means. R package version 1.8.4-1. https://cran.r-project.org/package=emmeans (15 December 2025, date last accessed).

[skaf461-B26] Liu C. , Cui Y., Li X., Yao M. 2021a. Microeco: an R package for data mining in microbial community ecology. FEMS. Microbiol. Ecol. 97(2):fiaa255. doi:10.1093/femsec/fiaa255 33332530

[skaf461-B27] Liu C. , Li X., Mansoldo F. R. P., An J., Kou Y., Zhang X., Wang J., Zeng J., Vermelho A. B., Yao M. 2022. Microbial habitat specificity largely affects microbial co-occurrence patterns and functional profiles in wetland soils. Geoderma. 418:115866. doi:10.1016/j.geoderma.2022.115866

[skaf461-B28] Liu G. , Li P., Hou L., Niu Q., Pu G., Wang B., Du T., Kim S. W., Niu P., Li Q. et al. 2021b. Metagenomic analysis reveals new microbiota related to fiber digestion in pigs. Front. Microbiol. 12:746717. doi:10.3389/fmicb.2021.746717 34867862 PMC8637618

[skaf461-B29] Love M. I. , Huber W., Anders S. 2014. Moderated estimation of fold change and dispersion for RNA-seq data with DESeq2. Genome Biol. 15(12):550. doi:10.1186/s13059-014-0550-8 25516281 PMC4302049

[skaf461-B30] Mach N. , Berri M., Estellé J., Levenez F., Lemonnier G., Denis C., Leplat J. J., Chevaleyre C., Billon Y., Doré J. et al. 2015. Early-life establishment of the swine gut microbiome and impact on host phenotypes. Environ. Microbiol. Rep. 7(3):554–569. doi:10.1111/1758-2229.12285 25727666

[skaf461-B31] Martínez-Porchas M. , Villalpando-Canchola E., Vargas-Albores F. 2016. Significant loss of sensitivity and specificity in the taxonomic classification occurs when short 16S rRNA gene sequences are used. Heliyon. 2(9):e00170. doi:10.1016/j.heliyon.2016.e00170 27699286 PMC5037269

[skaf461-B32] McCormack U. M. , Curião T., Buzoianu S. G., Prieto M. L., Ryan T., Varley P., Crispie F., Magowan E., Metzler-Zebeli B. U., Berry D. et al. 2017. Exploring a possible link between the intestinal microbiota and feed efficiency in pigs. Appl. Environ. Microbiol. 83(15):e00380-17. doi:10.1128/AEM.00380-17 28526795 PMC5514681

[skaf461-B33] McCormack U. M. , Curião T., Metzler-Zebeli B. U., Magowan E., Berry D. P., Reyer H., Prieto M. L., Buzoianu S. G., Harrison M., Rebeiz N. et al. 2019. Porcine feed efficiency-associated intestinal microbiota and physiological traits: finding consistent cross-locational biomarkers for residual feed intake. mSystems. 4(4):e00324-18. doi:10.1128/msystems.00324-18 31213524 PMC6581691

[skaf461-B34] McMurdie P. J. , Holmes S. 2013. Phyloseq: an R package for reproducible interactive analysis and graphics of microbiome census data. PLoS One. 8(4):e61217. doi:10.1371/journal.pone.0061217 23630581 PMC3632530

[skaf461-B35] Mikkelsen L. , Højberg O., Jensen B. 2007. Coarse structured feed stimulates members of the genera *Lactobacillus* and *Mitsuokella* as well as propionate and butyrate producers in the pig stomach. Livest. Sci. 109:153–156. doi:10.1016/j.livsci.2007.01.130

[skaf461-B36] Mikkelsen L. L. , Naughton P. J., Hedemann M. S., Jensen B. B. 2004. Effects of physical properties of feed on microbial ecology and survival of *Salmonella enterica* serovar typhimurium in the pig gastrointestinal tract. Appl. Environ. Microbiol. 70(6):3485–3492. doi:10.1128/AEM.70.6.3485-3492.2004 15184147 PMC427765

[skaf461-B37] Oksanen , Blanchet J. F., G., Friendly M., Kindt R., Legendre P., McGlinn D., Minchin P. R., O’Hara R. B., Simpson G. L., Solymos P. et al. 2020. Vegan: Community Ecology Package. R package version 2.6-4. https://cran.r-project.org/package=vegan (15 December 2025, date last accessed).

[skaf461-B38] O’Meara F. M. , Gardiner G. E., O’Doherty J. V., Lawlor P. G. 2020a. The effect of feed form and delivery method on feed microbiology and growth performance in grow-finisher pigs. J. Anim. Sci. 98:1–11. doi:10.1093/jas/skaa021 PMC7205396 31957788

[skaf461-B39] O’Meara F. M. , Gardiner G. E., Clarke D., Cummins W., O’Doherty J. V., Lawlor P. G. 2020b. Microbiological assessment of liquid feed for finisher pigs on commercial pig units. J. Appl. Microbiol. 130:356–369. doi:10.1111/jam.14785 32681565

[skaf461-B40] O’Meara F. M. , Gardiner G. E., O’Doherty J. V., Clarke D., Cummins W., Lawlor P. G. 2020c. Effect of wet/dry, fresh liquid, fermented whole diet liquid and fermented cereal liquid feeding on feed microbial quality and growth in grow-finisher pigs. J. Anim. Sci. 98:1–36. doi:10.1093/jas/skaa166 PMC7299551 32441755

[skaf461-B41] Quan J. , Cai G., Yang M., Zeng Z., Ding R., Wang X., Zhuang Z., Zhou S., Li S., Yang H. et al. 2019. Exploring the fecal microbial composition and metagenomic functional capacities associated with feed efficiency in commercial DLY pigs. Front. Microbiol. 10:52. doi:10.3389/fmicb.2019.00052 30761104 PMC6361760

[skaf461-B42] Quan J. , Cai G., Ye J., Yang M., Ding R., Wang X., Zheng E., Fu D., Li S., Zhou S. et al. 2018. A global comparison of the microbiome compositions of three gut locations in commercial pigs with extreme feed conversion ratios. Sci. Rep. 8(1):4536. doi:10.1038/s41598-018-22692-0 29540768 PMC5852056

[skaf461-B43] Quan J. , Wu Z., Ye Y., Peng L., Wu J., Ruan D., Qiu Y., Ding R., Wang X., Zheng E. et al. 2020. Metagenomic characterization of intestinal regions in pigs with contrasting feed efficiency. Front. Microbiol. 11:32. doi:10.3389/fmicb.2020.00032 32038603 PMC6989599

[skaf461-B44] Quast C. , Pruesse E., Yilmaz P., Gerken J., Schweer T., Yarza P., Peplies J., Glöckner F. O. 2013. The SILVA ribosomal RNA gene database project: improved data processing and web-based tools. Nucleic. Acids. Res. 41(Database issue):D590–D596. doi:10.1093/nar/gks1219 23193283 PMC3531112

[skaf461-B45] Russell P. J. , Geary T. M., Brooks P. H., Campbell A. 1996. Performance, water use and effluent output of weaner pigs fed *ad libitum* with either dry pellets or liquid feed and the role of microbial activity in the liquid feed. J. Sci. Food. Agric. 72:8–16. doi:10.1002/(SICI)1097-0010(199609)72:1<8::AID-JSFA646>3.0.CO;2-K

[skaf461-B46] Segata N. , Izard J., Waldron L., Gevers D., Miropolsky L., Garrett W. S., Huttenhower C. 2011. Metagenomic biomarker discovery and explanation. Genome. Biol. 12(6):R60. doi:10.1186/gb-2011-12-6-r60 21702898 PMC3218848

[skaf461-B47] Si J. , Feng L., Gao J., Huang Y., Zhang G., Mo J., Zhu S., Qi W., Liang J., Lan G. 2020. Evaluating the association between feed efficiency and the fecal microbiota of early-life duroc pigs using 16S rRNA sequencing. AMB. Express. 10(1):115. doi:10.1186/s13568-020-01050-2 32562009 PMC7305293

[skaf461-B48] Sitthicharoenchai P. , Burrough E. R., Arruda B. L., Sahin O., dos Santos J. G., Magstadt D. R., Piñeyro P. E., Schwartz K. J., Rahe M. C. 2022. *Streptococcus gallolyticus* and bacterial endocarditis in swine, United States, 2015–2020. Emerg. Infect. Dis. 28(1):192–195. doi:10.3201/eid2801.210998 34932445 PMC8714216

[skaf461-B49] Tan Z. , Yang T., Wang Y., Xing K., Zhang F., Zhao X., Ao H., Chen S., Liu J., Wang C. 2017. Metagenomic analysis of cecal microbiome identified microbiota and functional capacities associated with feed efficiency in landrace finishing pigs. Front. Microbiol. 8:1546–1511. doi:10.3389/fmicb.2017.01546 28848539 PMC5554500

[skaf461-B50] Torres-Pitarch A. , Gardiner G. E., Cormican P., Rea M., Crispie F., O’Doherty J. V., Cozannet P., Ryan T., Cullen J., Lawlor P. G. 2020a. Effect of cereal fermentation and carbohydrase supplementation on growth, nutrient digestibility and intestinal microbiota in liquid-fed grow-finishing pigs. Sci. Rep. 10(1):13716. doi:10.1038/s41598-020-70443-x 32792575 PMC7426827

[skaf461-B51] Torres-Pitarch A. , Gardiner G. E., Cormican P., Rea M., Crispie F., O’Doherty J. V., Cozannet P., Ryan T., Lawlor P. G. 2020b. Effect of cereal soaking and carbohydrase supplementation on growth, nutrient digestibility and intestinal microbiota in liquid-fed grow-finishing pigs. Sci. Rep. 10(1):1023. doi:10.1038/s41598-020-57668-6 31974415 PMC6978375

[skaf461-B52] Vukmirović R. , Čolović, Rakita S., Brlek T., Đuragić O., Solà-Oriol D. 2017. Importance of feed structure (particle size) and feed form (mash vs. pellets) in pig nutrition—a review. Anim. Feed. Sci. Technol. 233:133–144. doi:10.1016/j.anifeedsci.2017.06.016

[skaf461-B53] Wang X. , Tsai T., Deng F., Wei X., Chai J., Knapp J., Apple J., Maxwell C. V., Lee J. A., Li Y. et al. 2019. Longitudinal investigation of the swine gut microbiome from birth to market reveals stage and growth performance associated bacteria. Microbiome. 7(1):109–118. doi:10.1186/s40168-019-0721-7 31362781 PMC6664762

[skaf461-B54] Wickham H. 2016. ggplot2: Elegant graphics for data analysis. 2nd ed. Springer International Publishing, Cham, Switzerland. doi:10.1080/15366367.2019.1565254 (15 December 2025, date last accessed).

[skaf461-B55] Yang H. , Huang X., Fang S., He M., Zhao Y., Wu Z., Yang M., Zhang Z., Chen C., Huang L. 2017. Unraveling the fecal micro­biota and metagenomic functional capacity associated with feed ­efficiency in pigs. Front. Microbiol. 8:1555–1511. doi:10.3389/fmicb.2017.01555 28861066 PMC5559535

[skaf461-B56] Yang H. , Yang M., Fang S., Huang X., He M., Ke S., Gao J., Wu J., Zhou Y., Fu H. et al. 2018. Evaluating the profound effect of gut microbiome on host appetite in pigs. BMC. Microbiol. 18(1):215. doi:10.1186/s12866-018-1364-8 30547751 PMC6295093

