# Supplementary material for: Profiling the bacteriome of a diet fed in meal or pelleted form, delivered as dry, wet/dry, or liquid feed and its impact on the fecal and intestinal bacteriome of grow-finisher pigs
Source: J Anim Sci. 2026 Jan 9;104:skaf461. doi: 10.1093/jas/skaf461 (PMC12903947; doi:10.1093/jas/skaf461)
Supplement: skaf461_Supplementary_Data [file skaf461_supplementary_data.pdf]

Supplemental Material

Profiling the bacteriome of a diet fed in meal or pelleted form, delivered as dry, wet/dry or liquid feed and its impact on the fecal and intestinal bacteriome of grow-finisher pigs

James T. Cullen,[*,†] Peadar G. Lawlor,[†] Paul Cormican,[‡] Gillian E. Gardiner[*,1]

[*]Eco-Innovation Research Centre, Department of Science, South East Technological University, Waterford, X91K0EK, Ireland

[†]Teagasc Pig Development Department, Animal and Grassland Research and Innovation Centre, Moorepark, Fermoy, County Cork, P61C996, Ireland

[‡]Animal and Bioscience Research Department, Animal and Grassland Research and Innovation Centre, Teagasc Grange, Dunsany, County Meath, C15 PW93, Ireland

[1]Corresponding author: Gillian.Gardiner@setu.ie

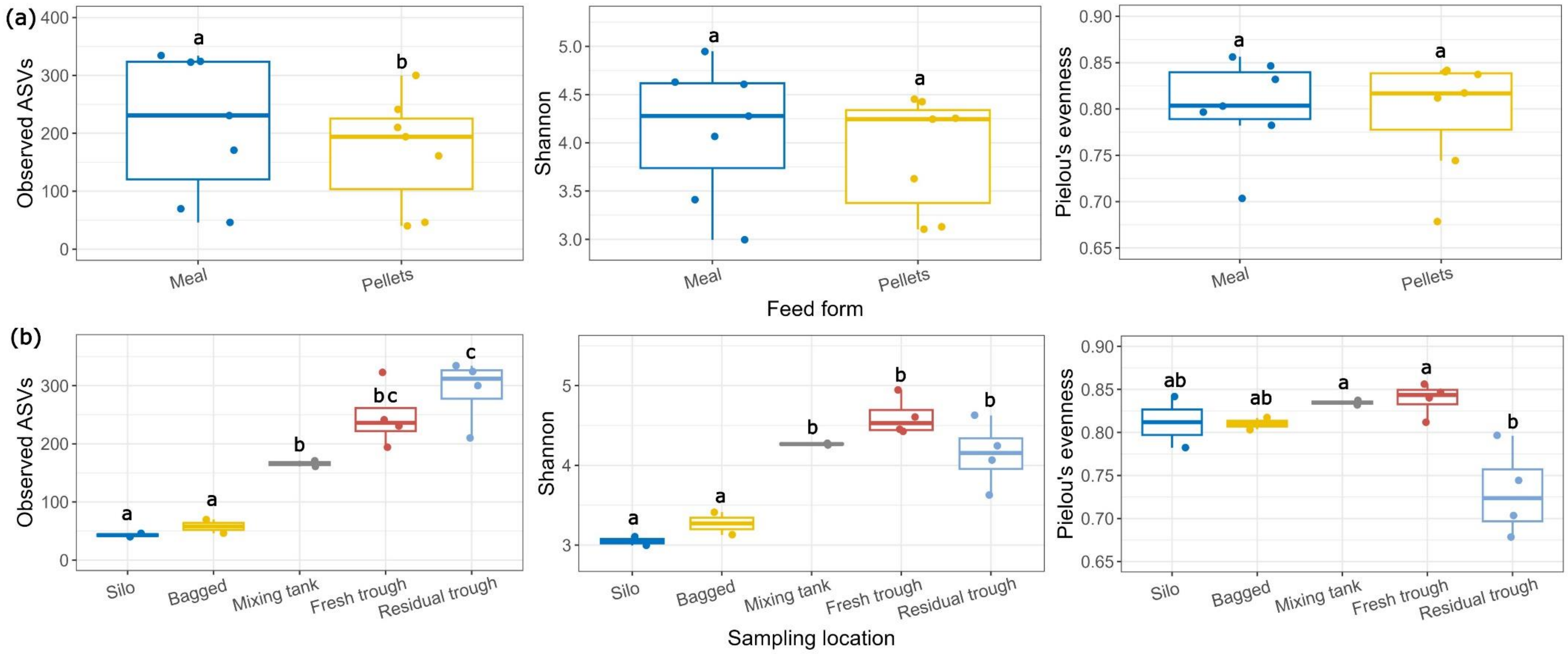

Supplementary Figure S1: Boxplots displaying alpha-diversity [Observed amplicon sequence variants (ASVs), Shannon diversity and Pielou's evenness] of the bacteriome of the experimental diets on day 27 of the experiment by **(a)** feed form (meal; n =7, or pellets; n = 7) and **(b)** sampling location. Dry feed collected from the silo (n = 2) was used to prepare the liquid diets while the bagged dry feed (n = 2) was used for the dry and wet/dry diets. The mixing tank (n = 2), fresh trough (n = 4) and residual trough (n = 4) samples were collected from the liquid feed treatments. For each individual boxplot, feed forms or sampling locations that do not share a common letter are significantly different ($P \leq 0.05$).

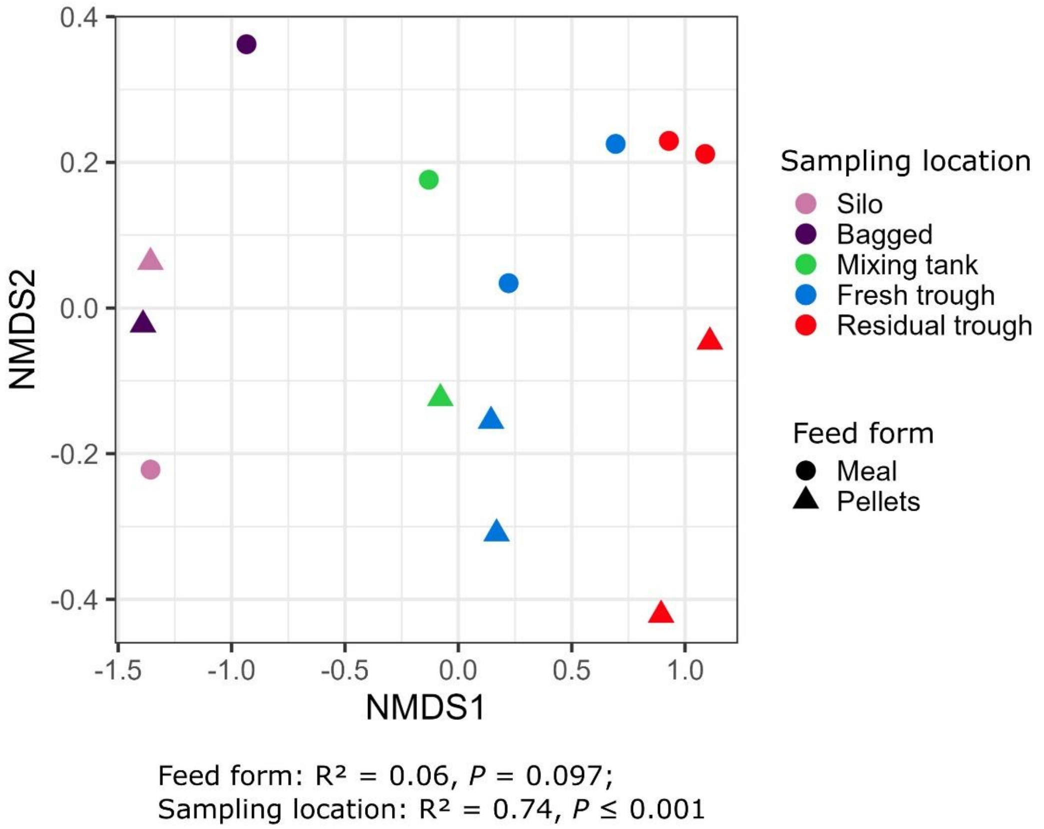

Supplementary Figure S2: Non-metric multidimensional scaling (NMDS) plots based on Bray-Curtis dissimilarity in feed collected on day 27 of the experiment by sampling location and feed form (meal or pellets). Dry feed collected from the silo (*n* = 2) was used to prepare the liquid diets while the bagged feed (*n* = 2) was used for the dry and wet/dry diets. The mixing tank (*n* = 2), fresh trough (*n* = 4) and residual trough (*n* = 4) samples were collected from the liquid feed treatments. Permutational analysis of variance (PERMANOVA) results for the feed samples are presented below each respective plot.

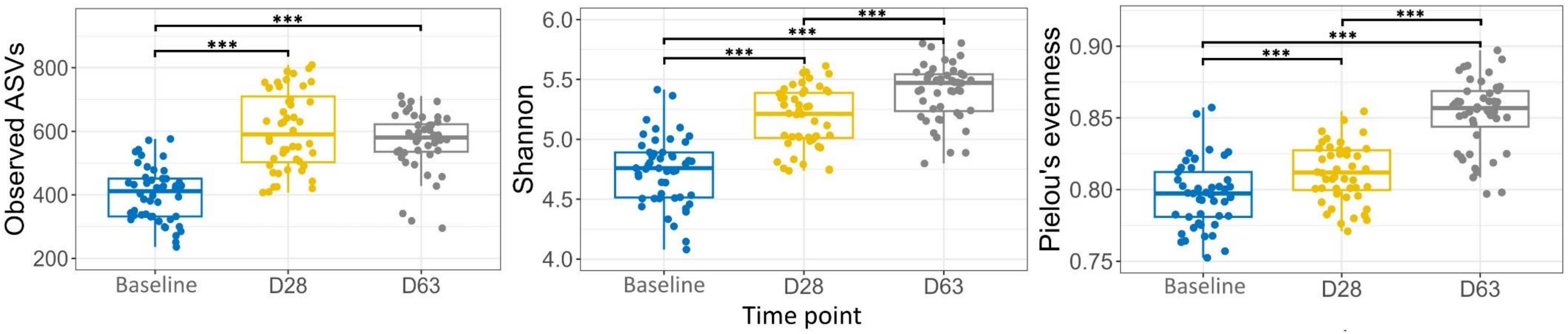

Supplementary Figure S3: Boxplots displaying alpha-diversity [Observed amplicon sequence variants (ASVs), Shannon diversity and Pielou's evenness] of the fecal bacteriome of grow-finisher pigs fed dry, liquid or wet/dry feed in meal or pelleted form at baseline, on day 28 (D28) and day 63 (D63) of the experiment. Data are averaged across feed forms and delivery methods for each time point, except at baseline where all diets were fed in meal form. *** $P < 0.001$.

(a)

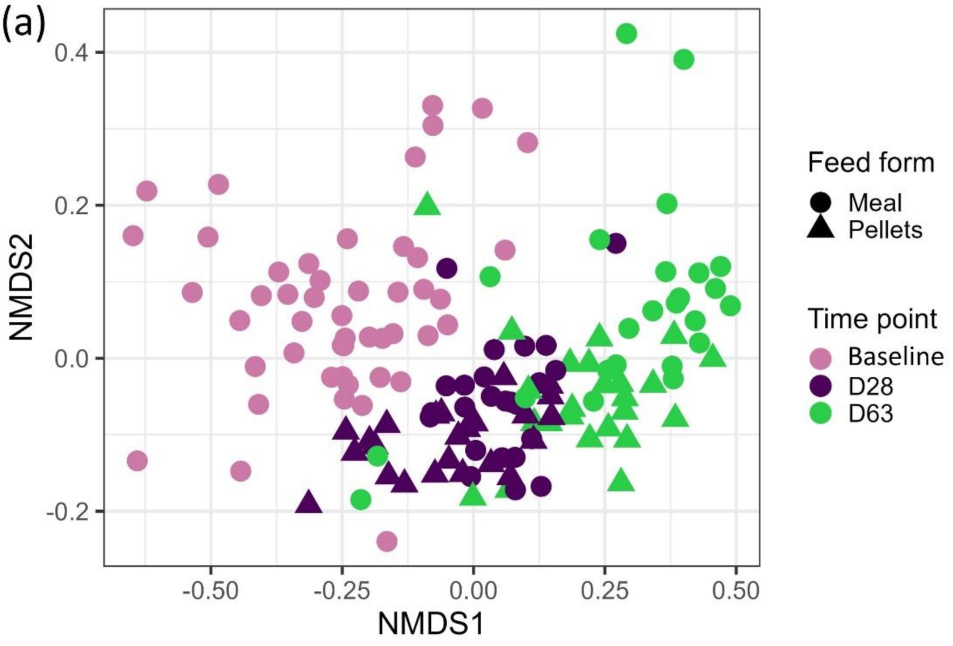

Time point: $R^2 = 0.22$, $P \leq 0.001$;
Feed form: $R^2 = 0.03$, $P \leq 0.001$;
Delivery method: $R^2 = 0.02$, $P \leq 0.01$

(b)

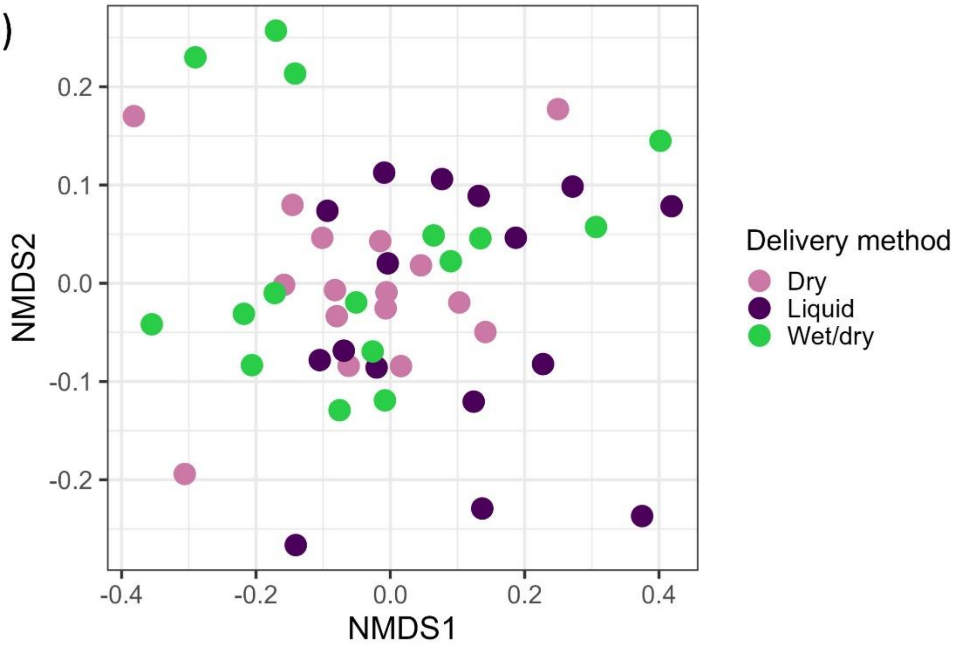

Delivery method: $R^2 = 0.07$, $P \leq 0.01$

Supplementary Figure S4: Non-metric multidimensional scaling (NMDS) plots based on Bray-Curtis dissimilarity in the feces of grow-finisher pigs **(a)** by feed form and time point at baseline (*n* = 48), day 28 (D28; *n* = 48) and day 63 (D63; *n* = 48), and **(b)** by delivery method at baseline (*n* = 48) where all diets were fed in meal form. Permutational analysis of variance (PERMANOVA) results for the fecal samples are presented below each respective plot.

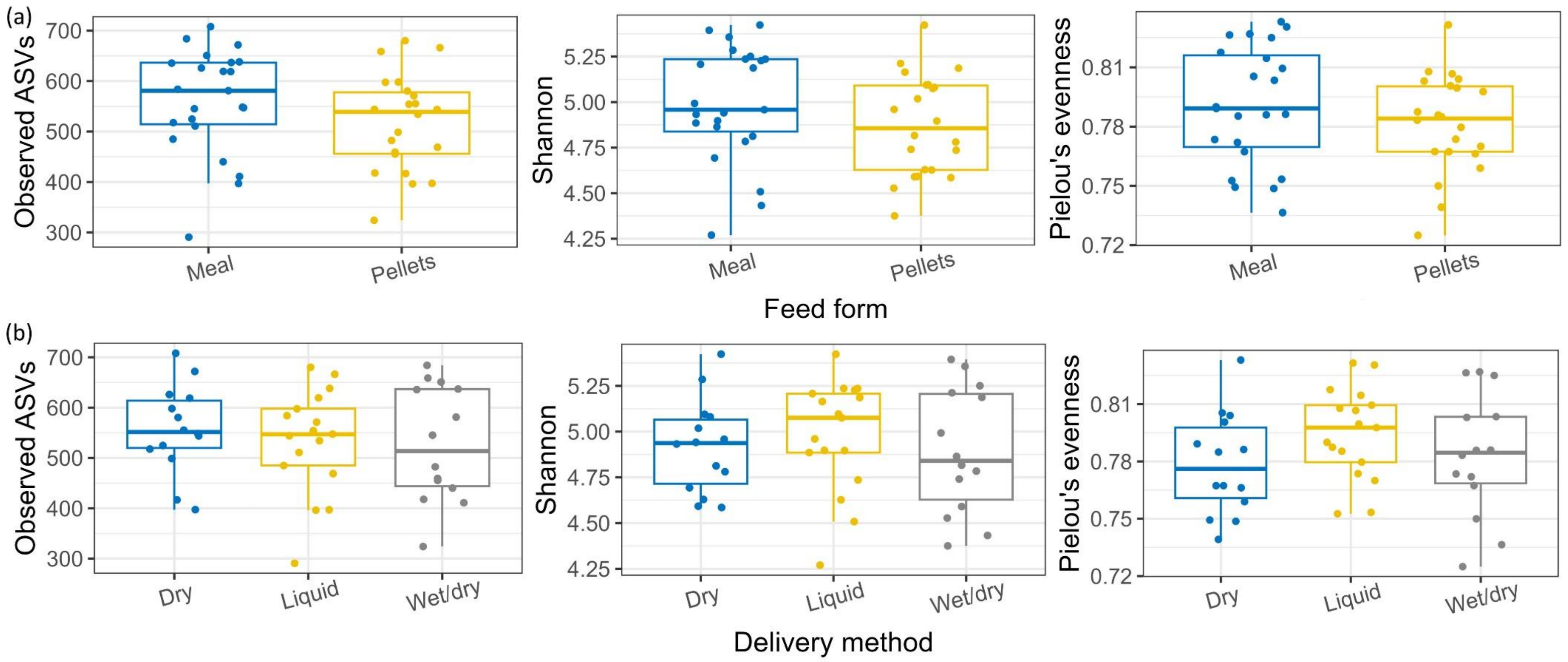

Supplementary Figure S5: Boxplots displaying alpha-diversity [Observed amplicon sequence variants (ASVs), Shannon diversity and Pielou's evenness] of the bacteriome in the caecal digesta of grow-finisher pigs fed dry, liquid or wet/dry feed in meal or pelleted form. **(a)** Alpha-diversity by feed form (meal; *n* = 23, or pellets; *n* = 22), averaged across delivery methods. **(b)** Alpha-diversity by delivery method (dry; *n* = 14, liquid; *n* = 17, or wet/dry; *n* =14), averaged across feed forms.

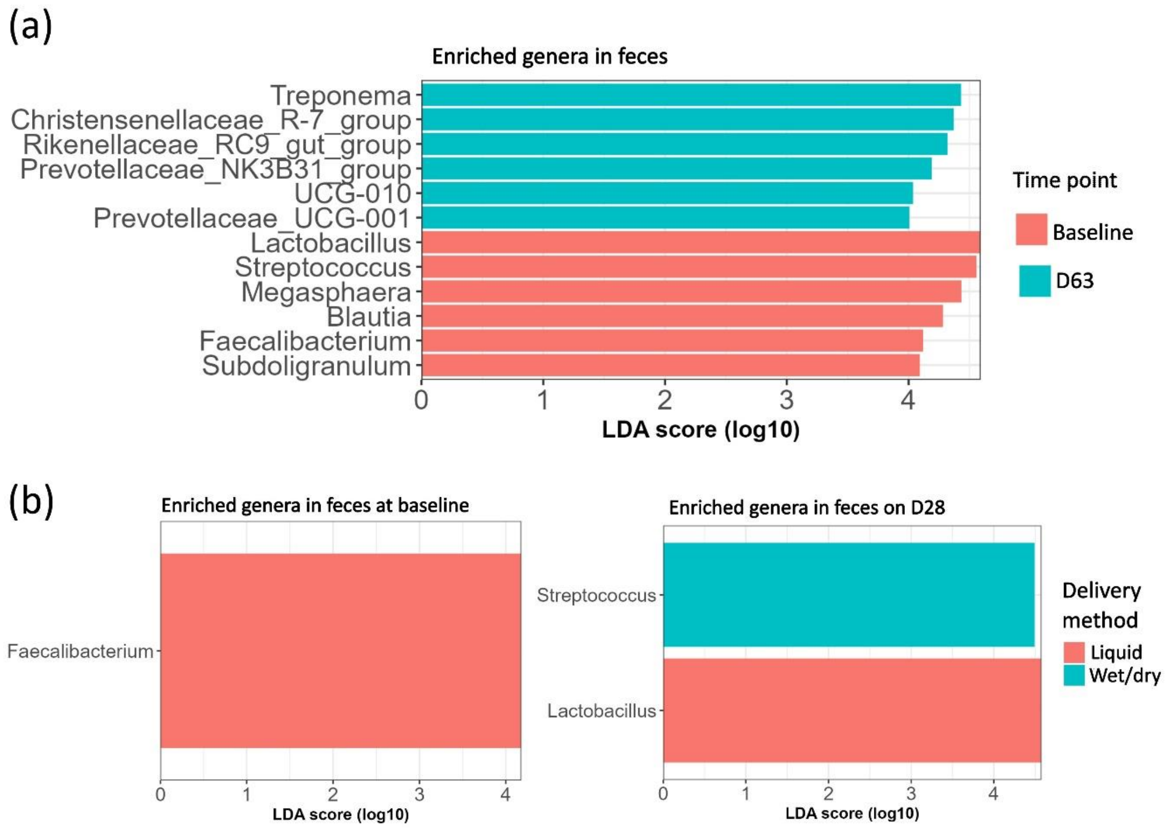

Supplementary Figure S6: Differentially abundant bacterial genera in the feces of grow-finisher pigs fed dry, liquid or wet/dry feed in meal or pelleted form by **(a)** time point [baseline; *n* = 48 and day 63 (D63); *n* = 48], and **(b)** between delivery methods (dry; *n* =16, liquid; *n* =16, or wet/dry; *n* = 16) at baseline and on day 28 (D28). Note that at baseline all diets were fed in meal form. Differential abundances between time points and delivery methods were identified by linear discriminant analysis (LDA) with effect size (LEfSe). Only genera with an LDA score ($\log_{10}$) > 4.0 are shown.

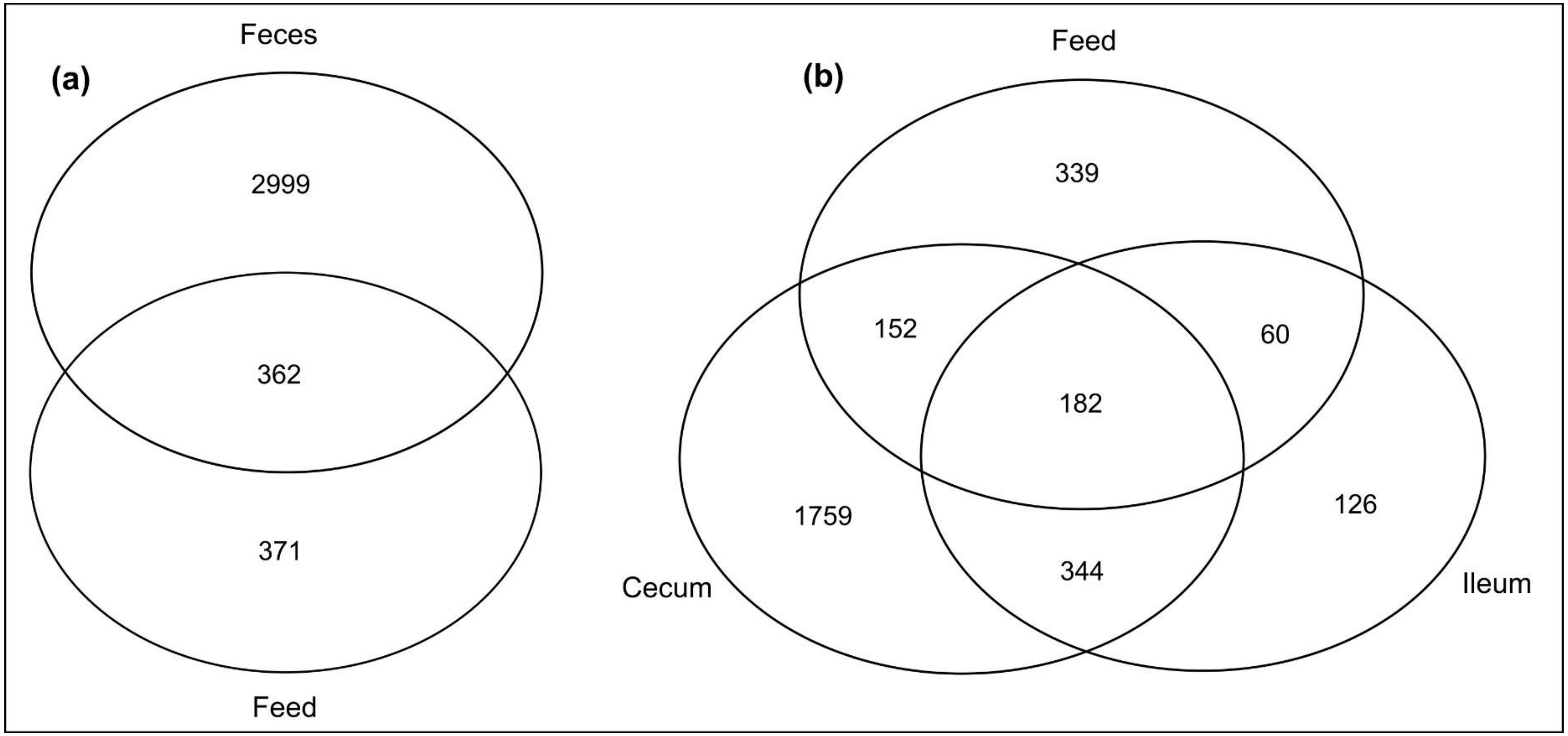

Supplementary Figure S7: Venn diagrams displaying the number of shared bacterial amplicon sequence variants (ASVs) **(a)** between feed and fecal samples and **(b)** between feed and intestinal digesta samples.

Supplementary Table S1: Composition of the experimental diet (on an as-fed basis, g/kg unless otherwise stated)[1] (O'Meara et al. 2020a).

**Ingredient composition**[2]	
Wheat	400.0
Barley	382.7
Soya bean meal	183.0
Limestone flour	11.0
Soya oil	9.7
Lysine HCl	3.8
Salt	3.0
L-Threonine	1.7
Celite	2.0
Vitamin and mineral premix[3]	1.0
Mono dicalcium phosphate	1.0
DL-Methionine	0.9
L-Tryptophan	0.2
Phytase[4]	0.1
**Chemical composition**	
Dry matter	877.0
Crude protein	174.0
Ash	39.2
Neutral detergent fiber	163.3
Gross energy, Mcal/kg	3.82
Lysine	10.6
Methionine	4.3
Threonine	7.2
Digestible energy, Mcal/kg[2]	3.30
Net energy, Mcal/kg[2]	2.34

Oil[2]	25.7
SID lysine[2]	10.0
Total calcium[2]	6.6
Total phosphorus[2]	2.6

[1]Values are the mean of experimental diets from experiment 1 and experiment 2 (O'Meara et al. 2020a).
[2]Calculated values.
[3]Vitamin and mineral premix provided per kilogram of complete diet: Cu from copper sulphate, 15 mg; Fe from ferrous sulphate monohydrate, 24 mg; Mn from manganese oxide, 31 mg; Zn from zinc oxide, 80 mg; I from potassium iodate, 0.3 mg; Se from sodium selenite, 0.2 mg; retinyl acetate, 0.7 mg; cholecalciferol, 12.7 µg; DL-alpha-tocopheryl acetate, 40 mg; vitamin K, 4 mg; vitamin B12, 15 µg; riboflavin, 2 mg; nicotinic acid, 12 mg; pantothenic acid, 10 mg; vitamin B1, 2 mg; vitamin B6, 3 mg; and celite 2,000 mg/kg.
[4]The diet contained 500 phytase units (FYT) per kilogram feed from RONOZYME HiPhos (DSM, Belfast, UK).

Supplementary Table S2: Bacterial genera present at ≥ 1 % mean relative abundance (RA, %) by treatment in the ileal and caecal digesta and feces of grow-finisher pigs fed the experimental diets.

Genus	Sample type	Treatment	Mean RA
Streptococcus	Ileum	Wet/dry pellets	36.90
Clostridium sensu strico 1	Ileum	Liquid pellets	34.20
Clostridium sensu strico 1	Ileum	Wet/dry meal	30.00
Clostridium sensu strico 1	Ileum	Dry meal	29.10
Streptococcus	Ileum	Dry pellets	28.90
Clostridium sensu strico 1	Ileum	Dry pellets	27.60
Clostridium sensu strico 1	Ileum	Wet/dry pellets	26.70
Streptococcus	Ileum	Liquid pellets	25.10
Lactobacillus	Ileum	Liquid meal	24.50
Clostridium sensu strico 1	Ileum	Liquid meal	23.10
Streptococcus	Ileum	Wet/dry meal	22.80
Terrisporobacter	Ileum	Wet/dry meal	16.10
Terrisporobacter	Ileum	Liquid pellets	16.00
Streptococcus	Ileum	Dry meal	15.20
Lactobacillus	Ileum	Dry pellets	13.50
Escherichia-Shigella	Ileum	Dry pellets	12.20
Terrisporobacter	Ileum	Dry meal	11.90
Terrisporobacter	Ileum	Liquid meal	11.20
Terrisporobacter	Ileum	Wet/dry pellets	10.90
Escherichia-Shigella	Ileum	Wet/dry pellets	9.70
Terrisporobacter	Ileum	Dry pellets	9.60
Megasphaera	Ileum	Liquid meal	9.40
Mitsuokella	Ileum	Dry meal	8.70
Streptococcus	Ileum	Liquid meal	8.50
Lactobacillus	Ileum	Wet/dry pellets	7.50
Megasphaera	Ileum	Dry meal	7.40
Lactobacillus	Ileum	Wet/dry meal	7.20
Lactobacillus	Ileum	Dry meal	6.50
Mitsuokella	Ileum	Liquid meal	6.10
Escherichia-Shigella	Ileum	Wet/dry meal	5.60
Prevotella	Ileum	Dry meal	5.40
Prevotella	Ileum	Liquid meal	5.20
Actinobacillus	Ileum	Liquid pellets	5.00
Escherichia-Shigella	Ileum	Liquid pellets	4.40
Turicibacter	Ileum	Wet/dry meal	3.90
Mitsuokella	Ileum	Wet/dry meal	3.70
Turicibacter	Ileum	Dry meal	3.40
Escherichia-Shigella	Ileum	Liquid meal	3.30

Escherichia-Shigella	Ileum	Dry meal	3.20
Actinobacillus	Ileum	Wet/dry meal	2.90
Romboutsia	Ileum	Liquid pellets	2.80
Turicibacter	Ileum	Liquid pellets	2.80
Actinobacillus	Ileum	Dry meal	2.80
Lactobacillus	Ileum	Liquid pellets	2.30
Turicibacter	Ileum	Dry pellets	2.10
Veillonella	Ileum	Wet/dry pellets	2.00
Romboutsia	Ileum	Wet/dry meal	1.90
Actinobacillus	Ileum	Wet/dry pellets	1.60
Veillonella	Ileum	Liquid pellets	1.60
Megasphaera	Ileum	Wet/dry meal	1.60
Turicibacter	Ileum	Wet/dry pellets	1.40
Romboutsia	Ileum	Dry pellets	1.40
Bifidobacterium	Ileum	Liquid meal	1.40
Romboutsia	Ileum	Wet/dry pellets	1.30
Romboutsia	Ileum	Dry meal	1.30
Actinobacillus	Ileum	Dry pellets	1.20
Turicibacter	Ileum	Liquid meal	1.20
Prevotella	Caecum	Dry pellets	20.36
Prevotella	Caecum	Wet/dry pellets	19.78
Prevotella	Caecum	Liquid pellets	18.93
Clostridium sensu strico 1	Caecum	Dry meal	17.53
Prevotella	Caecum	Liquid meal	16.95
Prevotella	Caecum	Wet/dry meal	15.94
Clostridium sensu strico 1	Caecum	Wet/dry meal	14.48
Prevotella	Caecum	Dry meal	13.90
Clostridium sensu strico 1	Caecum	Wet/dry pellets	12.86
Lactobacillus	Caecum	Liquid meal	12.82
Clostridium sensu strico 1	Caecum	Liquid pellets	12.46
Clostridium sensu strico 1	Caecum	Dry pellets	11.42
Clostridium sensu strico 1	Caecum	Liquid meal	9.34
Streptococcus	Caecum	Wet/dry pellets	6.68
Streptococcus	Caecum	Dry pellets	6.64
Prevotellaceae NK3B31 group	Caecum	Liquid meal	6.57
Terrisporobacter	Caecum	Dry meal	6.25
Prevotellaceae NK3B31 group	Caecum	Liquid pellets	6.11
Alloprevotella	Caecum	Liquid pellets	6.05
Terrisporobacter	Caecum	Liquid pellets	5.91
Prevotellaceae NK3B31 group	Caecum	Dry meal	5.79
Alloprevotella	Caecum	Wet/dry pellets	5.78
Alloprevotella	Caecum	Dry meal	5.45
Terrisporobacter	Caecum	Wet/dry meal	5.32
Prevotellaceae NK3B31 group	Caecum	Wet/dry meal	5.09

Alloprevotella	Caecum	Dry pellets	4.82
Alloprevotella	Caecum	Liquid meal	4.80
Alloprevotella	Caecum	Wet/dry meal	4.79
Streptococcus	Caecum	Wet/dry meal	4.55
Prevotellaceae NK3B31 group	Caecum	Wet/dry pellets	4.53
Terrisporobacter	Caecum	Wet/dry pellets	4.51
Streptococcus	Caecum	Liquid pellets	4.45
Terrisporobacter	Caecum	Dry pellets	4.43
Prevotellaceae NK3B31 group	Caecum	Dry pellets	4.37
Megasphaera	Caecum	Liquid meal	4.34
Megasphaera	Caecum	Wet/dry pellets	4.07
Megasphaera	Caecum	Dry pellets	4.05
Terrisporobacter	Caecum	Liquid meal	3.99
Lactobacillus	Caecum	Dry meal	3.79
Anaerovibrio	Caecum	Liquid meal	3.78
Lactobacillus	Caecum	Dry pellets	3.71
Anaerovibrio	Caecum	Liquid pellets	3.60
Lactobacillus	Caecum	Wet/dry pellets	3.58
Anaerovibrio	Caecum	Dry pellets	3.26
Streptococcus	Caecum	Dry meal	3.20
Subdoligranulum	Caecum	Dry pellets	3.20
Anaerovibrio	Caecum	Wet/dry pellets	3.11
Prevotellaceae UCG-003	Caecum	Wet/dry meal	2.84
Subdoligranulum	Caecum	Liquid pellets	2.81
Megasphaera	Caecum	Dry meal	2.80
Blautia	Caecum	Dry pellets	2.78
Anaerovibrio	Caecum	Wet/dry meal	2.73
Phascolarctobacterium	Caecum	Liquid pellets	2.73
Subdoligranulum	Caecum	Liquid meal	2.72
Phascolarctobacterium	Caecum	Wet/dry meal	2.67
Phascolarctobacterium	Caecum	Liquid meal	2.64
Subdoligranulum	Caecum	Wet/dry pellets	2.61
Anaerovibrio	Caecum	Dry meal	2.54
Phascolarctobacterium	Caecum	Dry pellets	2.46
Mitsuokella	Caecum	Dry meal	2.31
Blautia	Caecum	Wet/dry pellets	2.26
Rikenellaceae RC9 gut group	Caecum	Wet/dry meal	2.24
Blautia	Caecum	Liquid meal	2.14
Muribaculaceae	Caecum	Dry meal	2.14
Muribaculaceae	Caecum	Liquid meal	2.08
Blautia	Caecum	Liquid pellets	2.05
Blautia	Caecum	Wet/dry meal	2.03
Phascolarctobacterium	Caecum	Dry meal	2.01
Subdoligranulum	Caecum	Dry meal	1.98

Megasphaera	Caecum	Liquid pellets	1.94
Subdoligranulum	Caecum	Wet/dry meal	1.94
Escherichia-Shigella	Caecum	Dry pellets	1.94
Prevotellaceae UCG-003	Caecum	Dry meal	1.85
Phascolarctobacterium	Caecum	Wet/dry pellets	1.84
Muribaculaceae	Caecum	Wet/dry meal	1.83
Faecalibacterium	Caecum	Liquid meal	1.81
Rikenellaceae RC9 gut group	Caecum	Dry meal	1.80
Megasphaera	Caecum	Wet/dry meal	1.78
Faecalibacterium	Caecum	Dry pellets	1.78
Muribaculaceae	Caecum	Dry pellets	1.76
Campylobacter	Caecum	Dry meal	1.75
Streptococcus	Caecum	Liquid meal	1.66
Blautia	Caecum	Dry meal	1.65
Lactobacillus	Caecum	Wet/dry meal	1.65
Muribaculaceae	Caecum	Liquid pellets	1.64
Roseburia	Caecum	Liquid meal	1.61
Roseburia	Caecum	Dry pellets	1.57
Campylobacter	Caecum	Liquid pellets	1.55
Muribaculaceae	Caecum	Wet/dry pellets	1.54
UCG-005	Caecum	Dry meal	1.53
Roseburia	Caecum	Liquid pellets	1.51
Faecalibacterium	Caecum	Liquid pellets	1.50
Lactobacillus	Caecum	Liquid pellets	1.46
Prevotellaceae UCG-003	Caecum	Liquid meal	1.45
UCG-005	Caecum	Wet/dry meal	1.43
Turicibacter	Caecum	Dry meal	1.42
Prevotellaceae UCG-003	Caecum	Wet/dry pellets	1.42
Faecalibacterium	Caecum	Wet/dry pellets	1.41
Actinobacillus	Caecum	Dry meal	1.41
Campylobacter	Caecum	Wet/dry meal	1.40
Rikenellaceae RC9 gut group	Caecum	Liquid meal	1.38
Turicibacter	Caecum	Wet/dry meal	1.36
Rikenellaceae RC9 gut group	Caecum	Liquid pellets	1.35
Rikenellaceae RC9 gut group	Caecum	Dry pellets	1.34
Rikenellaceae RC9 gut group	Caecum	Wet/dry pellets	1.34
UCG-005	Caecum	Liquid meal	1.32
Faecalibacterium	Caecum	Wet/dry meal	1.32
Treponema	Caecum	Wet/dry meal	1.27
UCG-005	Caecum	Liquid pellets	1.27
Agathobacter	Caecum	Liquid meal	1.23
Prevotellaceae UCG-003	Caecum	Liquid pellets	1.23
Gastranaerophilales	Caecum	Liquid pellets	1.18
Mitsuokella	Caecum	Wet/dry pellets	1.17

Agathobacter	Caecum	Dry pellets	1.16
Turicibacter	Caecum	Liquid pellets	1.15
Agathobacter	Caecum	Liquid pellets	1.14
Roseburia	Caecum	Wet/dry meal	1.13
Dialister	Caecum	Wet/dry pellets	1.11
Escherichia-Shigella	Caecum	Liquid pellets	1.09
Mitsuokella	Caecum	Wet/dry meal	1.01
Agathobacter	Caecum	Wet/dry meal	1.01
Prevotella	Baseline feces	Dry meal	14.25
Prevotella	Baseline feces	Wet/dry meal	14.09
Prevotella	Baseline feces	Wet/dry pellets	13.95
Prevotella	Baseline feces	Dry pellets	13.66
Prevotella	Baseline feces	Liquid pellets	13.40
Prevotella	Baseline feces	Liquid meal	11.99
Lactobacillus	Baseline feces	Liquid pellets	11.77
Streptococcus	Baseline feces	Liquid meal	11.23
Lactobacillus	Baseline feces	Liquid meal	9.64
Streptococcus	Baseline feces	Liquid pellets	9.16
Streptococcus	Baseline feces	Dry pellets	9.06
Streptococcus	Baseline feces	Wet/dry pellets	8.85
Streptococcus	Baseline feces	Dry meal	8.63
Streptococcus	Baseline feces	Wet/dry meal	8.25
Lactobacillus	Baseline feces	Dry pellets	7.87
Lactobacillus	Baseline feces	Wet/dry pellets	7.86
Clostridium sensu strico 1	Baseline feces	Dry meal	7.74
Megasphaera	Baseline feces	Liquid pellets	6.95
Clostridium sensu strico 1	Baseline feces	Wet/dry pellets	6.89
Clostridium sensu strico 1	Baseline feces	Wet/dry meal	6.88
Lactobacillus	Baseline feces	Dry meal	6.61
Megasphaera	Baseline feces	Liquid meal	6.25
Megasphaera	Baseline feces	Wet/dry pellets	5.60
Megasphaera	Baseline feces	Wet/dry meal	5.51
Clostridium sensu strico 1	Baseline feces	Liquid pellets	5.50
Megasphaera	Baseline feces	Dry pellets	5.47
Prevotellaceae NK3B31 group	Baseline feces	Dry pellets	5.21
Prevotellaceae NK3B31 group	Baseline feces	Liquid meal	5.18
Muribaculaceae	Baseline feces	Dry meal	4.87
Prevotellaceae NK3B31 group	Baseline feces	Dry meal	4.87
Megasphaera	Baseline feces	Dry meal	4.76
Prevotellaceae NK3B31 group	Baseline feces	Wet/dry meal	4.70
Subdoligranulum	Baseline feces	Liquid pellets	4.47
Faecalibacterium	Baseline feces	Liquid pellets	4.40
Muribaculaceae	Baseline feces	Dry pellets	4.28
Lactobacillus	Baseline feces	Wet/dry meal	4.25

Blautia	Baseline feces	Wet/dry pellets	4.25
Muribaculaceae	Baseline feces	Wet/dry pellets	4.22
Blautia	Baseline feces	Liquid pellets	4.22
Anaerovibrio	Baseline feces	Dry meal	4.18
Clostridium sensu strico 1	Baseline feces	Liquid meal	4.17
Muribaculaceae	Baseline feces	Wet/dry meal	4.08
Clostridium sensu strico 1	Baseline feces	Dry pellets	4.03
Blautia	Baseline feces	Wet/dry meal	3.85
Prevotellaceae NK3B31 group	Baseline feces	Wet/dry pellets	3.75
Anaerovibrio	Baseline feces	Wet/dry meal	3.62
Blautia	Baseline feces	Dry pellets	3.54
Blautia	Baseline feces	Liquid meal	3.54
Muribaculaceae	Baseline feces	Liquid meal	3.44
Phascolarctobacterium	Baseline feces	Liquid pellets	3.34
Faecalibacterium	Baseline feces	Liquid meal	3.32
Phascolarctobacterium	Baseline feces	Wet/dry pellets	3.13
Phascolarctobacterium	Baseline feces	Dry pellets	3.12
Rikenellaceae RC9 gut group	Baseline feces	Dry meal	2.96
Muribaculaceae	Baseline feces	Liquid pellets	2.91
Rikenellaceae RC9 gut group	Baseline feces	Wet/dry meal	2.87
Blautia	Baseline feces	Dry meal	2.84
Rikenellaceae RC9 gut group	Baseline feces	Wet/dry pellets	2.80
Anaerovibrio	Baseline feces	Liquid meal	2.80
Rikenellaceae RC9 gut group	Baseline feces	Liquid meal	2.69
Phascolarctobacterium	Baseline feces	Wet/dry meal	2.63
Rikenellaceae RC9 gut group	Baseline feces	Dry pellets	2.54
Subdoligranulum	Baseline feces	Liquid meal	2.52
Subdoligranulum	Baseline feces	Wet/dry pellets	2.47
Prevotellaceae NK3B31 group	Baseline feces	Liquid pellets	2.35
Subdoligranulum	Baseline feces	Dry pellets	2.32
Subdoligranulum	Baseline feces	Wet/dry meal	2.24
Phascolarctobacterium	Baseline feces	Dry meal	2.19
Phascolarctobacterium	Baseline feces	Liquid meal	2.17
Roseburia	Baseline feces	Dry pellets	2.11
Succinivibrio	Baseline feces	Liquid pellets	2.07
Roseburia	Baseline feces	Dry meal	2.05
Roseburia	Baseline feces	Liquid pellets	2.00
Alloprevotella	Baseline feces	Wet/dry pellets	1.99
Rikenellaceae RC9 gut group	Baseline feces	Liquid pellets	1.96
Faecalibacterium	Baseline feces	Wet/dry meal	1.90
Subdoligranulum	Baseline feces	Dry meal	1.89
Terrisporobacter	Baseline feces	Wet/dry pellets	1.88
Catenibacterium	Baseline feces	Liquid pellets	1.84
Prevotellaceae UCG-003	Baseline feces	Wet/dry pellets	1.82

Faecalibacterium	Baseline feces	Wet/dry pellets	1.78
Anaerovibrio	Baseline feces	Wet/dry pellets	1.76
Anaerovibrio	Baseline feces	Dry pellets	1.76
Ruminococcus	Baseline feces	Wet/dry pellets	1.75
Ruminococcus	Baseline feces	Dry meal	1.74
Ruminococcus	Baseline feces	Wet/dry meal	1.72
Roseburia	Baseline feces	Wet/dry meal	1.68
Mitsuokella	Baseline feces	Liquid meal	1.67
Succinivibrio	Baseline feces	Dry pellets	1.67
Alloprevotella	Baseline feces	Wet/dry meal	1.62
Alloprevotella	Baseline feces	Liquid meal	1.58
Succinivibrio	Baseline feces	Wet/dry meal	1.58
Agathobacter	Baseline feces	Liquid meal	1.53
Faecalibacterium	Baseline feces	Dry pellets	1.53
Treponema	Baseline feces	Dry pellets	1.51
Prevotellaceae UCG-003	Baseline feces	Wet/dry meal	1.51
Alloprevotella	Baseline feces	Liquid pellets	1.47
Alloprevotella	Baseline feces	Dry pellets	1.46
Agathobacter	Baseline feces	Dry meal	1.45
Ruminococcus	Baseline feces	Dry pellets	1.43
Ruminococcus	Baseline feces	Liquid meal	1.43
Terrisporobacter	Baseline feces	Dry meal	1.42
Ruminococcus	Baseline feces	Liquid pellets	1.38
Roseburia	Baseline feces	Liquid meal	1.36
Prevotellaceae UCG-003	Baseline feces	Dry pellets	1.35
Terrisporobacter	Baseline feces	Wet/dry meal	1.28
Agathobacter	Baseline feces	Wet/dry meal	1.27
Faecalibacterium	Baseline feces	Dry meal	1.27
Dialister	Baseline feces	Liquid meal	1.27
Acidaminococcus	Baseline feces	Liquid meal	1.26
Agathobacter	Baseline feces	Liquid pellets	1.22
Terrisporobacter	Baseline feces	Liquid pellets	1.21
Acidaminococcus	Baseline feces	Liquid pellets	1.19
Roseburia	Baseline feces	Wet/dry pellets	1.18
Catenibacterium	Baseline feces	Dry pellets	1.14
Escherichia-Shigella	Baseline feces	Wet/dry pellets	1.13
Alloprevotella	Baseline feces	Dry meal	1.10
UCG-005	Baseline feces	Wet/dry pellets	1.10
Catenibacterium	Baseline feces	Liquid meal	1.09
Agathobacter	Baseline feces	Dry pellets	1.09
UCG-002	Baseline feces	Dry meal	1.07
Catenibacterium	Baseline feces	Wet/dry pellets	1.04
Escherichia-Shigella	Baseline feces	Dry pellets	1.04
Selenomonas	Baseline feces	Dry pellets	1.04

Mitsuokella	Baseline feces	Dry pellets	1.03
Prevotellaceae UCG-003	Baseline feces	Dry meal	1.02
UCG-005	Baseline feces	Wet/dry meal	1.02
Escherichia-Shigella	Baseline feces	Liquid pellets	1.01
Acidaminococcus	Baseline feces	Wet/dry pellets	1.01
Anaerovibrio	Baseline feces	Liquid pellets	1.01
Christensenellaceae R-7 group	Baseline feces	Wet/dry pellets	1.00
Prevotella	D28 feces	Wet/dry pellets	20.67
Prevotella	D28 feces	Liquid pellets	19.13
Prevotella	D28 feces	Dry pellets	17.31
Lactobacillus	D28 feces	Liquid meal	14.88
Prevotella	D28 feces	Dry meal	13.11
Prevotella	D28 feces	Liquid meal	12.10
Prevotella	D28 feces	Wet/dry meal	10.89
Streptococcus	D28 feces	Wet/dry pellets	10.67
Clostridium sensu strico 1	D28 feces	Wet/dry meal	10.14
Lactobacillus	D28 feces	Dry meal	8.92
Streptococcus	D28 feces	Dry pellets	8.86
Clostridium sensu strico 1	D28 feces	Dry meal	8.60
Clostridium sensu strico 1	D28 feces	Liquid pellets	8.43
Prevotellaceae NK3B31 group	D28 feces	Dry meal	7.75
Lactobacillus	D28 feces	Wet/dry meal	7.08
Prevotellaceae NK3B31 group	D28 feces	Wet/dry meal	7.04
Prevotellaceae NK3B31 group	D28 feces	Liquid meal	6.81
Megasphaera	D28 feces	Wet/dry pellets	6.79
Lactobacillus	D28 feces	Liquid pellets	6.44
Streptococcus	D28 feces	Wet/dry meal	6.12
Streptococcus	D28 feces	Liquid pellets	5.99
Clostridium sensu strico 1	D28 feces	Dry pellets	5.72
Prevotellaceae NK3B31 group	D28 feces	Liquid pellets	5.49
Megasphaera	D28 feces	Liquid meal	4.93
Clostridium sensu strico 1	D28 feces	Liquid meal	4.91
Clostridium sensu strico 1	D28 feces	Wet/dry pellets	4.86
Lactobacillus	D28 feces	Dry pellets	4.83
Megasphaera	D28 feces	Dry pellets	4.66
Prevotellaceae NK3B31 group	D28 feces	Dry pellets	4.56
Muribaculaceae	D28 feces	Liquid meal	4.49
Muribaculaceae	D28 feces	Wet/dry meal	4.43
Streptococcus	D28 feces	Dry meal	4.43
Muribaculaceae	D28 feces	Dry meal	4.26
Rikenellaceae RC9 gut group	D28 feces	Wet/dry meal	4.05
Rikenellaceae RC9 gut group	D28 feces	Liquid meal	3.97
Rikenellaceae RC9 gut group	D28 feces	Dry meal	3.91
Muribaculaceae	D28 feces	Liquid pellets	3.81

Muribaculaceae	D28 feces	Dry pellets	3.61
Megasphaera	D28 feces	Liquid pellets	3.59
Megasphaera	D28 feces	Wet/dry meal	3.42
Alloprevotella	D28 feces	Wet/dry meal	3.19
Rikenellaceae RC9 gut group	D28 feces	Dry pellets	3.16
Terrisporobacter	D28 feces	Wet/dry meal	3.15
Muribaculaceae	D28 feces	Wet/dry pellets	3.07
Alloprevotella	D28 feces	Dry meal	2.95
Terrisporobacter	D28 feces	Liquid pellets	2.80
Megasphaera	D28 feces	Dry meal	2.80
Treponema	D28 feces	Liquid meal	2.69
Alloprevotella	D28 feces	Liquid meal	2.65
Rikenellaceae RC9 gut group	D28 feces	Liquid pellets	2.58
Prevotellaceae NK3B31 group	D28 feces	Wet/dry pellets	2.48
Subdoligranulum	D28 feces	Liquid pellets	2.47
UCG-005	D28 feces	Liquid meal	2.45
Rikenellaceae RC9 gut group	D28 feces	Wet/dry pellets	2.44
Anaerovibrio	D28 feces	Liquid meal	2.40
Subdoligranulum	D28 feces	Liquid meal	2.37
Terrisporobacter	D28 feces	Dry meal	2.30
Subdoligranulum	D28 feces	Wet/dry pellets	2.28
Treponema	D28 feces	Dry meal	2.21
Subdoligranulum	D28 feces	Dry pellets	2.17
Blautia	D28 feces	Dry pellets	2.16
Lactobacillus	D28 feces	Wet/dry pellets	2.12
Alloprevotella	D28 feces	Liquid pellets	2.11
Dialister	D28 feces	Wet/dry pellets	2.10
Acidaminococcus	D28 feces	Wet/dry pellets	2.09
Christensenellaceae R-7 group	D28 feces	Liquid meal	2.07
Faecalibacterium	D28 feces	Wet/dry pellets	2.06
Subdoligranulum	D28 feces	Dry meal	2.04
Anaerovibrio	D28 feces	Dry meal	2.01
Phascolarctobacterium	D28 feces	Liquid meal	2.01
UCG-005	D28 feces	Dry meal	1.96
Phascolarctobacterium	D28 feces	Wet/dry meal	1.95
Anaerovibrio	D28 feces	Wet/dry meal	1.94
Alloprevotella	D28 feces	Dry pellets	1.93
UCG-005	D28 feces	Wet/dry meal	1.90
Phascolarctobacterium	D28 feces	Dry meal	1.86
Blautia	D28 feces	Wet/dry pellets	1.86
Blautia	D28 feces	Liquid pellets	1.80
Terrisporobacter	D28 feces	Wet/dry pellets	1.79
Anaerovibrio	D28 feces	Liquid pellets	1.77
Blautia	D28 feces	Liquid meal	1.76

Subdoligranulum	D28 feces	Wet/dry meal	1.74
Roseburia	D28 feces	Wet/dry pellets	1.73
Roseburia	D28 feces	Liquid meal	1.73
Prevotellaceae UCG-003	D28 feces	Liquid pellets	1.72
Christensenellaceae R-7 group	D28 feces	Wet/dry meal	1.69
Ruminococcus	D28 feces	Dry pellets	1.68
Alloprevotella	D28 feces	Wet/dry pellets	1.64
Phascolarctobacterium	D28 feces	Liquid pellets	1.63
Christensenellaceae R-7 group	D28 feces	Dry meal	1.61
Faecalibacterium	D28 feces	Dry pellets	1.57
Treponema	D28 feces	Dry pellets	1.56
Prevotellaceae UCG-003	D28 feces	Wet/dry meal	1.55
Blautia	D28 feces	Wet/dry meal	1.54
Christensenellaceae R-7 group	D28 feces	Dry pellets	1.54
Blautia	D28 feces	Dry meal	1.53
Prevotellaceae UCG-003	D28 feces	Dry meal	1.52
Phascolarctobacterium	D28 feces	Dry pellets	1.48
UCG-005	D28 feces	Liquid pellets	1.46
Anaerovibrio	D28 feces	Wet/dry pellets	1.46
Treponema	D28 feces	Wet/dry meal	1.44
UCG-005	D28 feces	Dry pellets	1.40
Ruminococcus	D28 feces	Wet/dry pellets	1.38
Ruminococcus	D28 feces	Wet/dry meal	1.36
Ruminococcus	D28 feces	Dry meal	1.35
Treponema	D28 feces	Liquid pellets	1.35
Prevotellaceae UCG-003	D28 feces	Liquid meal	1.34
Ruminococcus	D28 feces	Liquid pellets	1.33
Prevotellaceae UCG-003	D28 feces	Dry pellets	1.32
Terrisporobacter	D28 feces	Dry pellets	1.32
Anaerovibrio	D28 feces	Dry pellets	1.25
Roseburia	D28 feces	Dry meal	1.24
Mitsuokella	D28 feces	Wet/dry pellets	1.22
Ruminococcus	D28 feces	Liquid meal	1.22
Phascolarctobacterium	D28 feces	Wet/dry pellets	1.20
Terrisporobacter	D28 feces	Liquid meal	1.18
Roseburia	D28 feces	Dry pellets	1.18
Roseburia	D28 feces	Liquid pellets	1.10
Faecalibacterium	D28 feces	Liquid pellets	1.06
Dialister	D28 feces	Dry pellets	1.05
Coprococcus	D28 feces	Liquid meal	1.04
Roseburia	D28 feces	Wet/dry meal	1.03
UCG-010	D28 feces	Liquid pellets	1.03
Christensenellaceae R-7 group	D28 feces	Liquid pellets	1.02
UCG-005	D28 feces	Wet/dry pellets	1.01

Streptococcus	D28 feces	Liquid meal	1.01
Faecalibacterium	D28 feces	Liquid meal	1.00
Prevotella	D63 feces	Liquid pellets	19.48
Prevotella	D63 feces	Liquid meal	14.52
Prevotella	D63 feces	Wet/dry pellets	14.40
Prevotella	D63 feces	Dry pellets	11.68
Clostridium sensu strico 1	D63 feces	Wet/dry meal	9.71
Prevotellaceae NK3B31 group	D63 feces	Dry pellets	9.42
Rikenellaceae RC9 gut group	D63 feces	Wet/dry meal	8.91
Clostridium sensu strico 1	D63 feces	Dry meal	8.78
Prevotellaceae NK3B31 group	D63 feces	Liquid pellets	8.77
Prevotellaceae NK3B31 group	D63 feces	Dry meal	7.34
Treponema	D63 feces	Wet/dry pellets	7.09
Christensenellaceae R-7 group	D63 feces	Wet/dry meal	6.78
Rikenellaceae RC9 gut group	D63 feces	Dry meal	6.33
Lactobacillus	D63 feces	Liquid meal	6.23
Christensenellaceae R-7 group	D63 feces	Dry meal	6.13
Prevotella	D63 feces	Dry meal	6.09
Prevotellaceae NK3B31 group	D63 feces	Liquid meal	6.00
Muribaculaceae	D63 feces	Dry meal	5.71
Treponema	D63 feces	Dry meal	5.70
Muribaculaceae	D63 feces	Dry pellets	5.66
Rikenellaceae RC9 gut group	D63 feces	Wet/dry pellets	5.44
Clostridium sensu strico 1	D63 feces	Dry pellets	5.34
Rikenellaceae RC9 gut group	D63 feces	Dry pellets	5.33
Prevotellaceae NK3B31 group	D63 feces	Wet/dry meal	5.23
Treponema	D63 feces	Wet/dry meal	5.04
Muribaculaceae	D63 feces	Wet/dry pellets	5.01
Treponema	D63 feces	Liquid pellets	4.86
Rikenellaceae RC9 gut group	D63 feces	Liquid pellets	4.83
Muribaculaceae	D63 feces	Liquid pellets	4.80
Muribaculaceae	D63 feces	Wet/dry meal	4.76
Rikenellaceae RC9 gut group	D63 feces	Liquid meal	4.76
Prevotellaceae NK3B31 group	D63 feces	Wet/dry pellets	4.58
Streptococcus	D63 feces	Wet/dry pellets	4.51
Clostridium sensu strico 1	D63 feces	Wet/dry pellets	4.50
Muribaculaceae	D63 feces	Liquid meal	4.41
Treponema	D63 feces	Dry pellets	4.40
Streptococcus	D63 feces	Dry pellets	4.26
Streptococcus	D63 feces	Liquid meal	4.24
Clostridium sensu strico 1	D63 feces	Liquid meal	4.17
Megasphaera	D63 feces	Liquid meal	4.06
Christensenellaceae R-7 group	D63 feces	Dry pellets	3.85
Christensenellaceae R-7 group	D63 feces	Liquid meal	3.79

Prevotella	D63 feces	Wet/dry meal	3.76
Clostridium sensu strico 1	D63 feces	Liquid pellets	3.55
Christensenellaceae R-7 group	D63 feces	Wet/dry pellets	3.43
Streptococcus	D63 feces	Liquid pellets	3.36
Alloprevotella	D63 feces	Liquid pellets	3.03
Prevotellaceae UCG-001	D63 feces	Wet/dry meal	2.88
Alloprevotella	D63 feces	Dry meal	2.69
Treponema	D63 feces	Liquid meal	2.69
UCG-010	D63 feces	Wet/dry pellets	2.60
Bacteroidales RF16 group	D63 feces	Dry meal	2.56
Prevotellaceae UCG-001	D63 feces	Wet/dry pellets	2.55
Alloprevotella	D63 feces	Wet/dry meal	2.54
Prevotellaceae UCG-003	D63 feces	Wet/dry meal	2.51
Alloprevotella	D63 feces	Dry pellets	2.48
Streptococcus	D63 feces	Dry meal	2.46
UCG-005	D63 feces	Wet/dry meal	2.34
Prevotellaceae UCG-003	D63 feces	Dry meal	2.32
Alloprevotella	D63 feces	Wet/dry pellets	2.24
Parabacteroides	D63 feces	Wet/dry meal	2.24
Terrisporobacter	D63 feces	Wet/dry meal	2.22
Prevotellaceae UCG-003	D63 feces	Liquid pellets	2.17
UCG-010	D63 feces	Dry pellets	2.14
UCG-010	D63 feces	Wet/dry meal	2.03
Phascolarctobacterium	D63 feces	Liquid pellets	2.02
UCG-010	D63 feces	Liquid pellets	2.00
Terrisporobacter	D63 feces	Dry meal	1.99
Christensenellaceae R-7 group	D63 feces	Liquid pellets	1.95
UCG-005	D63 feces	Dry meal	1.93
Prevotellaceae UCG-003	D63 feces	Dry pellets	1.91
dgA-11 gut group	D63 feces	Wet/dry pellets	1.90
Anaerovibrio	D63 feces	Liquid meal	1.89
Prevotellaceae UCG-003	D63 feces	Wet/dry pellets	1.88
Alloprevotella	D63 feces	Liquid meal	1.85
Megasphaera	D63 feces	Dry pellets	1.79
UCG-010	D63 feces	Dry meal	1.78
UCG-005	D63 feces	Liquid pellets	1.78
Terrisporobacter	D63 feces	Liquid pellets	1.71
Terrisporobacter	D63 feces	Dry pellets	1.68
Parabacteroides	D63 feces	Dry pellets	1.68
UCG-002	D63 feces	Dry meal	1.67
Terrisporobacter	D63 feces	Wet/dry pellets	1.66
Clostridia vadinBB60 group	D63 feces	Wet/dry pellets	1.65
UCG-005	D63 feces	Dry pellets	1.65
Phascolarctobacterium	D63 feces	Dry pellets	1.64

UCG-002	D63 feces	Wet/dry meal	1.63
Clostridia vadinBB60 group	D63 feces	Dry pellets	1.62
Terrisporobacter	D63 feces	Liquid meal	1.59
Lactobacillus	D63 feces	Dry pellets	1.55
UCG-005	D63 feces	Wet/dry pellets	1.53
Bacteroidales RF16 group	D63 feces	Dry pellets	1.53
Phascolarctobacterium	D63 feces	Liquid meal	1.53
UCG-002	D63 feces	Wet/dry pellets	1.48
Phascolarctobacterium	D63 feces	Dry meal	1.47
Bacteroidales RF16 group	D63 feces	Wet/dry meal	1.46
Ruminococcus	D63 feces	Wet/dry pellets	1.45
UCG-005	D63 feces	Liquid meal	1.45
Clostridia vadinBB60 group	D63 feces	Wet/dry meal	1.42
Streptococcus	D63 feces	Wet/dry meal	1.42
Lachnospiraceae XPB1014 group	D63 feces	Wet/dry meal	1.40
Phascolarctobacterium	D63 feces	Wet/dry meal	1.39
Parabacteroides	D63 feces	Dry meal	1.39
Lactobacillus	D63 feces	Dry meal	1.39
Phascolarctobacterium	D63 feces	Wet/dry pellets	1.38
p-2534-18B5 gut group	D63 feces	Liquid meal	1.37
Fibrobacter	D63 feces	Wet/dry pellets	1.36
Prevotellaceae UCG-001	D63 feces	Dry pellets	1.32
UCG-002	D63 feces	Liquid pellets	1.32
NK4A214 group	D63 feces	Wet/dry meal	1.31
Ruminococcus	D63 feces	Liquid meal	1.31
Clostridia vadinBB60 group	D63 feces	Dry meal	1.29
Megasphaera	D63 feces	Wet/dry pellets	1.29
Prevotellaceae UCG-003	D63 feces	Liquid meal	1.28
Anaerovibrio	D63 feces	Liquid pellets	1.27
UCG-002	D63 feces	Dry pellets	1.25
Lactobacillus	D63 feces	Wet/dry meal	1.24
WCHB1-41	D63 feces	Wet/dry pellets	1.22
Prevotellaceae UCG-001	D63 feces	Liquid pellets	1.20
Eubacterium coprostanoligenes group	D63 feces	Wet/dry pellets	1.18
p-2534-18B5 gut group	D63 feces	Dry meal	1.16
p-2534-18B5 gut group	D63 feces	Wet/dry meal	1.15
Clostridia UCG-014	D63 feces	Wet/dry meal	1.15
Anaerovibrio	D63 feces	Dry meal	1.15
Ruminococcus	D63 feces	Dry meal	1.14
WCHB1-41	D63 feces	Wet/dry meal	1.13
dgA-11 gut group	D63 feces	Dry meal	1.11
Ruminococcus	D63 feces	Liquid pellets	1.09
Prevotellaceae UCG-001	D63 feces	Liquid meal	1.09
UCG-010	D63 feces	Liquid meal	1.09

Subdoligranulum	D63 feces	Liquid pellets	1.09
Anaerovibrio	D63 feces	Dry pellets	1.08
Eubacterium ruminantium group	D63 feces	Liquid meal	1.08
WCHB1-41	D63 feces	Dry pellets	1.06
Parabacteroides	D63 feces	Wet/dry pellets	1.06
Parabacteroides	D63 feces	Liquid pellets	1.06
Family XIII AD3011 group	D63 feces	Wet/dry meal	1.06
Ruminococcus	D63 feces	Dry pellets	1.06
Clostridia vadinBB60 group	D63 feces	Liquid meal	1.05
Sphaerochaeta	D63 feces	Dry pellets	1.04
Eubacterium coprostanoligenes group	D63 feces	Wet/dry meal	1.04
Prevotellaceae UCG-001	D63 feces	Dry meal	1.03
p-1088-a5 gut group	D63 feces	Wet/dry meal	1.02
Bacteroides	D63 feces	Wet/dry meal	1.02
Megasphaera	D63 feces	Liquid pellets	1.01
Eubacterium coprostanoligenes group	D63 feces	Dry meal	1.00
NK4A214 group	D63 feces	Dry meal	1.00
Eubacterium coprostanoligenes group	D63 feces	Dry pellets	1.00

Supplementary Table S3: Bacterial genera ≥ 1 % mean relative abundance (RA, %) by feed form in the intestinal digesta and feces of grow-finisher pigs fed the experimental diets.

Genus	Sample	Feed Form	Mean RA
Clostridium sensu strico 1	Ileum	Pellets	30.02
Streptococcus	Ileum	Pellets	29.52
Clostridium sensu strico 1	Ileum	Meal	27.36
Streptococcus	Ileum	Meal	15.52
Terrisporobacter	Ileum	Meal	13.12
Lactobacillus	Ileum	Meal	12.98
Terrisporobacter	Ileum	Pellets	12.55
Escherichia-Shigella	Ileum	Pellets	8.32
Lactobacillus	Ileum	Pellets	7.25
Megasphaera	Ileum	Meal	6.06
Mitsuokella	Ileum	Meal	6.05
Escherichia-Shigella	Ileum	Meal	4.07
Prevotella	Ileum	Meal	3.60
Actinobacillus	Ileum	Pellets	2.86
Turicibacter	Ileum	Meal	2.79
Turicibacter	Ileum	Pellets	2.18
Actinobacillus	Ileum	Meal	2.04
Romboutsia	Ileum	Pellets	1.97
Romboutsia	Ileum	Meal	1.35
Veillonella	Ileum	Pellets	1.29
Prevotella	Caecum	Pellets	19.62
Prevotella	Caecum	Meal	15.67
Clostridium sensu strico 1	Caecum	Meal	13.62
Clostridium sensu strico 1	Caecum	Pellets	12.24
Lactobacillus	Caecum	Meal	6.19
Prevotellaceae NK3B31 group	Caecum	Meal	5.82
Streptococcus	Caecum	Pellets	5.75
Alloprevotella	Caecum	Pellets	5.59
Terrisporobacter	Caecum	Meal	5.14
Prevotellaceae NK3B31 group	Caecum	Pellets	5.13
Terrisporobacter	Caecum	Pellets	5.05
Alloprevotella	Caecum	Meal	4.99
Anaerovibrio	Caecum	Pellets	3.36
Megasphaera	Caecum	Pellets	3.19
Streptococcus	Caecum	Meal	3.13
Anaerovibrio	Caecum	Meal	3.03
Megasphaera	Caecum	Meal	2.98
Subdoligranulum	Caecum	Pellets	2.88
Lactobacillus	Caecum	Pellets	2.76
Phascolarctobacterium	Caecum	Meal	2.46

Phascolarctobacterium	Caecum	Pellets	2.40
Blautia	Caecum	Pellets	2.34
Subdoligranulum	Caecum	Meal	2.22
Prevotellaceae UCG-003	Caecum	Meal	2.06
Muribaculaceae	Caecum	Meal	2.01
Blautia	Caecum	Meal	1.95
Rikenellaceae RC9 gut group	Caecum	Meal	1.81
Muribaculaceae	Caecum	Pellets	1.65
Faecalibacterium	Caecum	Pellets	1.56
UCG-005	Caecum	Meal	1.42
Mitsuokella	Caecum	Meal	1.38
Roseburia	Caecum	Pellets	1.37
Faecalibacterium	Caecum	Meal	1.37
Rikenellaceae RC9 gut group	Caecum	Pellets	1.35
Escherichia-Shigella	Caecum	Pellets	1.32
Prevotellaceae UCG-003	Caecum	Pellets	1.20
Roseburia	Caecum	Meal	1.17
Campylobacter	Caecum	Meal	1.12
Agathobacter	Caecum	Pellets	1.09
UCG-005	Caecum	Pellets	1.08
Turicibacter	Caecum	Meal	1.04
Prevotella	D28 feces	Pellets	19.04
Prevotella	D28 feces	Meal	12.03
Lactobacillus	D28 feces	Meal	10.29
Streptococcus	D28 feces	Pellets	8.51
Clostridium sensu strico 1	D28 feces	Meal	7.88
Prevotellaceae NK3B31 group	D28 feces	Meal	7.20
Clostridium sensu strico 1	D28 feces	Pellets	6.34
Megasphaera	D28 feces	Pellets	5.01
Lactobacillus	D28 feces	Pellets	4.47
Muribaculaceae	D28 feces	Meal	4.40
Prevotellaceae NK3B31 group	D28 feces	Pellets	4.18
Rikenellaceae RC9 gut group	D28 feces	Meal	3.98
Streptococcus	D28 feces	Meal	3.85
Megasphaera	D28 feces	Meal	3.72
Muribaculaceae	D28 feces	Pellets	3.50
Alloprevotella	D28 feces	Meal	2.93
Rikenellaceae RC9 gut group	D28 feces	Pellets	2.73
Subdoligranulum	D28 feces	Pellets	2.31
Terrisporobacter	D28 feces	Meal	2.21
Anaerovibrio	D28 feces	Meal	2.12
Treponema	D28 feces	Meal	2.11
UCG-005	D28 feces	Meal	2.10
Subdoligranulum	D28 feces	Meal	2.05

Terrisporobacter	D28 feces	Pellets	1.97
Blautia	D28 feces	Pellets	1.94
Phascolarctobacterium	D28 feces	Meal	1.94
Alloprevotella	D28 feces	Pellets	1.89
Christensenellaceae R-7 group	D28 feces	Meal	1.79
Blautia	D28 feces	Meal	1.61
Faecalibacterium	D28 feces	Pellets	1.56
Anaerovibrio	D28 feces	Pellets	1.49
Prevotellaceae UCG-003	D28 feces	Meal	1.47
Ruminococcus	D28 feces	Pellets	1.46
Phascolarctobacterium	D28 feces	Pellets	1.44
Dialister	D28 feces	Pellets	1.35
Roseburia	D28 feces	Pellets	1.34
Roseburia	D28 feces	Meal	1.33
Ruminococcus	D28 feces	Meal	1.31
UCG-005	D28 feces	Pellets	1.29
Prevotellaceae UCG-003	D28 feces	Pellets	1.28
Treponema	D28 feces	Pellets	1.25
Acidaminococcus	D28 feces	Pellets	1.12
Christensenellaceae R-7 group	D28 feces	Pellets	1.09
Prevotella	D63 feces	Pellets	15.19
Prevotella	D63 feces	Meal	8.12
Prevotellaceae NK3B31 group	D63 feces	Pellets	7.59
Clostridium sensu strico 1	D63 feces	Meal	7.55
Rikenellaceae RC9 gut group	D63 feces	Meal	6.67
Prevotellaceae NK3B31 group	D63 feces	Meal	6.19
Christensenellaceae R-7 group	D63 feces	Meal	5.57
Treponema	D63 feces	Pellets	5.45
Rikenellaceae RC9 gut group	D63 feces	Pellets	5.20
Muribaculaceae	D63 feces	Pellets	5.16
Muribaculaceae	D63 feces	Meal	4.96
Treponema	D63 feces	Meal	4.48
Clostridium sensu strico 1	D63 feces	Pellets	4.47
Streptococcus	D63 feces	Pellets	4.04
Christensenellaceae R-7 group	D63 feces	Pellets	3.08
Lactobacillus	D63 feces	Meal	2.95
Streptococcus	D63 feces	Meal	2.71
Alloprevotella	D63 feces	Pellets	2.58
Alloprevotella	D63 feces	Meal	2.36
UCG-010	D63 feces	Pellets	2.25
Prevotellaceae UCG-003	D63 feces	Meal	2.04
Prevotellaceae UCG-003	D63 feces	Pellets	1.99
Terrisporobacter	D63 feces	Meal	1.94
UCG-005	D63 feces	Meal	1.90

Prevotellaceae UCG-001	D63 feces	Pellets	1.69
Terrisporobacter	D63 feces	Pellets	1.68
Phascolarctobacterium	D63 feces	Pellets	1.68
Prevotellaceae UCG-001	D63 feces	Meal	1.67
UCG-005	D63 feces	Pellets	1.65
UCG-010	D63 feces	Meal	1.63
Parabacteroides	D63 feces	Meal	1.49
Bacteroidales RF16 group	D63 feces	Meal	1.48
Megasphaera	D63 feces	Meal	1.47
Phascolarctobacterium	D63 feces	Meal	1.47
UCG-002	D63 feces	Meal	1.42
Clostridia vadinBB60 group	D63 feces	Pellets	1.41
Megasphaera	D63 feces	Pellets	1.36
UCG-002	D63 feces	Pellets	1.35
Parabacteroides	D63 feces	Pellets	1.27
Clostridia vadinBB60 group	D63 feces	Meal	1.25
p-2534-18B5 gut group	D63 feces	Meal	1.22
Ruminococcus	D63 feces	Pellets	1.20
dgA-11 gut group	D63 feces	Pellets	1.17
Ruminococcus	D63 feces	Meal	1.14
Lactobacillus	D63 feces	Pellets	1.12
Anaerovibrio	D63 feces	Meal	1.10
Anaerovibrio	D63 feces	Pellets	1.07
Lachnospiraceae XPB1014 group	D63 feces	Meal	1.02
WCHB1-41	D63 feces	Pellets	1.01
Eubacterium coprostanoligenes group	D63 feces	Pellets	1.00

Supplementary Table S4: Bacterial genera ≥ 1 % mean relative abundance (RA, %) across delivery methods in the intestinal digesta and feces of grow-finisher pigs fed the experimental diets.

Genus	Sample type	Delivery Method	Mean RA
Clostridium sensu strico 1	Ileum	Liquid	28.98
Streptococcus	Ileum	Wet/dry	28.84
Clostridium sensu strico 1	Ileum	Wet/dry	28.59
Clostridium sensu strico 1	Ileum	Dry	28.34
Streptococcus	Ileum	Dry	22.02
Streptococcus	Ileum	Liquid	17.3
Terrisporobacter	Ileum	Wet/dry	13.89
Terrisporobacter	Ileum	Liquid	13.73
Lactobacillus	Ileum	Liquid	12.73
Terrisporobacter	Ileum	Dry	10.72
Lactobacillus	Ileum	Dry	9.99
Escherichia-Shigella	Ileum	Dry	7.7
Escherichia-Shigella	Ileum	Wet/dry	7.33
Lactobacillus	Ileum	Wet/dry	7.29
Megasphaera	Ileum	Liquid	4.45
Mitsuokella	Ileum	Dry	4.39
Escherichia-Shigella	Ileum	Liquid	3.9
Megasphaera	Ileum	Dry	3.79
Prevotella	Ileum	Dry	3.1
Mitsuokella	Ileum	Liquid	2.9
Actinobacillus	Ileum	Liquid	2.9
Turicibacter	Ileum	Wet/dry	2.85
Prevotella	Ileum	Liquid	2.82
Turicibacter	Ileum	Dry	2.72
Actinobacillus	Ileum	Wet/dry	2.34
Mitsuokella	Ileum	Wet/dry	2.13
Turicibacter	Ileum	Liquid	2.01
Actinobacillus	Ileum	Dry	1.98
Romboutsia	Ileum	Liquid	1.9
Romboutsia	Ileum	Wet/dry	1.65
Romboutsia	Ileum	Dry	1.35
Veillonella	Ileum	Wet/dry	1.1
Veillonella	Ileum	Liquid	1.07
Prevotella	Caecum	Liquid	17.99
Prevotella	Caecum	Wet/dry	17.59
Prevotella	Caecum	Dry	17.13
Clostridium sensu strico 1	Caecum	Dry	14.47
Clostridium sensu strico 1	Caecum	Wet/dry	13.78
Clostridium sensu strico 1	Caecum	Liquid	11.00

Lactobacillus	Caecum	Liquid	6.81
Prevotellaceae NK3B31 group	Caecum	Liquid	6.33
Streptococcus	Caecum	Wet/dry	5.46
Alloprevotella	Caecum	Liquid	5.46
Terrisporobacter	Caecum	Dry	5.34
Alloprevotella	Caecum	Wet/dry	5.21
Alloprevotella	Caecum	Dry	5.14
Prevotellaceae NK3B31 group	Caecum	Dry	5.08
Terrisporobacter	Caecum	Liquid	5.00
Terrisporobacter	Caecum	Wet/dry	4.97
Streptococcus	Caecum	Dry	4.92
Prevotellaceae NK3B31 group	Caecum	Wet/dry	4.85
Lactobacillus	Caecum	Dry	3.75
Anaerovibrio	Caecum	Liquid	3.68
Megasphaera	Caecum	Dry	3.42
Streptococcus	Caecum	Liquid	3.14
Megasphaera	Caecum	Liquid	3.07
Anaerovibrio	Caecum	Dry	2.90
Anaerovibrio	Caecum	Wet/dry	2.89
Subdoligranulum	Caecum	Liquid	2.77
Megasphaera	Caecum	Wet/dry	2.76
Phascolarctobacterium	Caecum	Liquid	2.68
Subdoligranulum	Caecum	Dry	2.59
Lactobacillus	Caecum	Wet/dry	2.48
Phascolarctobacterium	Caecum	Wet/dry	2.32
Phascolarctobacterium	Caecum	Dry	2.23
Prevotellaceae UCG-003	Caecum	Wet/dry	2.23
Subdoligranulum	Caecum	Wet/dry	2.23
Blautia	Caecum	Dry	2.22
Blautia	Caecum	Wet/dry	2.13
Blautia	Caecum	Liquid	2.09
Muribaculaceae	Caecum	Dry	1.95
Rikenellaceae RC9 gut group	Caecum	Wet/dry	1.85
Muribaculaceae	Caecum	Liquid	1.85
Muribaculaceae	Caecum	Wet/dry	1.70
Faecalibacterium	Caecum	Liquid	1.64
Mitsuokella	Caecum	Dry	1.60
Rikenellaceae RC9 gut group	Caecum	Dry	1.57
Roseburia	Caecum	Liquid	1.56
Prevotellaceae UCG-003	Caecum	Dry	1.41
Rikenellaceae RC9 gut group	Caecum	Liquid	1.37
Faecalibacterium	Caecum	Wet/dry	1.36
Faecalibacterium	Caecum	Dry	1.35
Prevotellaceae UCG-003	Caecum	Liquid	1.33

UCG-005	Caecum	Liquid	1.29
UCG-005	Caecum	Dry	1.25
UCG-005	Caecum	Wet/dry	1.22
Escherichia-Shigella	Caecum	Dry	1.18
Agathobacter	Caecum	Liquid	1.18
Roseburia	Caecum	Dry	1.14
Mitsuokella	Caecum	Wet/dry	1.08
Roseburia	Caecum	Wet/dry	1.05
Gastranaerophilales	Caecum	Liquid	1.03
Turicibacter	Caecum	Wet/dry	1.01
Prevotella	Baseline feces	Wet/dry	14.02
Prevotella	Baseline feces	Dry	13.96
Prevotella	Baseline feces	Liquid	12.7
Lactobacillus	Baseline feces	Liquid	10.7
Streptococcus	Baseline feces	Liquid	10.19
Streptococcus	Baseline feces	Dry	8.84
Streptococcus	Baseline feces	Wet/dry	8.55
Lactobacillus	Baseline feces	Dry	7.24
Clostridium sensu strico 1	Baseline feces	Wet/dry	6.88
Megasphaera	Baseline feces	Liquid	6.6
Lactobacillus	Baseline feces	Wet/dry	6.06
Clostridium sensu strico 1	Baseline feces	Dry	5.88
Megasphaera	Baseline feces	Wet/dry	5.55
Megasphaera	Baseline feces	Dry	5.12
Prevotellaceae NK3B31 group	Baseline feces	Dry	5.04
Clostridium sensu strico 1	Baseline feces	Liquid	4.84
Muribaculaceae	Baseline feces	Dry	4.58
Prevotellaceae NK3B31 group	Baseline feces	Wet/dry	4.23
Muribaculaceae	Baseline feces	Wet/dry	4.15
Blautia	Baseline feces	Wet/dry	4.05
Blautia	Baseline feces	Liquid	3.88
Faecalibacterium	Baseline feces	Liquid	3.86
Prevotellaceae NK3B31 group	Baseline feces	Liquid	3.76
Subdoligranulum	Baseline feces	Liquid	3.49
Blautia	Baseline feces	Dry	3.19
Muribaculaceae	Baseline feces	Liquid	3.18
Anaerovibrio	Baseline feces	Dry	2.97
Phascolarctobacterium	Baseline feces	Wet/dry	2.88
Rikenellaceae RC9 gut group	Baseline feces	Wet/dry	2.83
Phascolarctobacterium	Baseline feces	Liquid	2.76
Rikenellaceae RC9 gut group	Baseline feces	Dry	2.75
Anaerovibrio	Baseline feces	Wet/dry	2.69
Phascolarctobacterium	Baseline feces	Dry	2.66
Subdoligranulum	Baseline feces	Wet/dry	2.36

Rikenellaceae RC9 gut group	Baseline feces	Liquid	2.33
Subdoligranulum	Baseline feces	Dry	2.1
Roseburia	Baseline feces	Dry	2.08
Anaerovibrio	Baseline feces	Liquid	1.9
Faecalibacterium	Baseline feces	Wet/dry	1.84
Alloprevotella	Baseline feces	Wet/dry	1.8
Ruminococcus	Baseline feces	Wet/dry	1.73
Roseburia	Baseline feces	Liquid	1.68
Prevotellaceae UCG-003	Baseline feces	Wet/dry	1.66
Ruminococcus	Baseline feces	Dry	1.59
Terrisporobacter	Baseline feces	Wet/dry	1.58
Alloprevotella	Baseline feces	Liquid	1.53
Catenibacterium	Baseline feces	Liquid	1.47
Roseburia	Baseline feces	Wet/dry	1.43
Ruminococcus	Baseline feces	Liquid	1.4
Faecalibacterium	Baseline feces	Dry	1.4
Agathobacter	Baseline feces	Liquid	1.38
Succinivibrio	Baseline feces	Liquid	1.36
Alloprevotella	Baseline feces	Dry	1.28
Agathobacter	Baseline feces	Dry	1.27
Mitsuokella	Baseline feces	Liquid	1.26
Acidaminococcus	Baseline feces	Liquid	1.22
Succinivibrio	Baseline feces	Dry	1.22
Prevotellaceae UCG-003	Baseline feces	Dry	1.19
Terrisporobacter	Baseline feces	Dry	1.14
Terrisporobacter	Baseline feces	Liquid	1.1
Escherichia-Shigella	Baseline feces	Wet/dry	1.06
UCG-005	Baseline feces	Wet/dry	1.06
Treponema	Baseline feces	Dry	1.05
Agathobacter	Baseline feces	Wet/dry	1.03
Prevotella	D28 feces	Wet/dry	15.78
Prevotella	D28 feces	Liquid	15.62
Prevotella	D28 feces	Dry	15.21
Lactobacillus	D28 feces	Liquid	10.66
Streptococcus	D28 feces	Wet/dry	8.39
Clostridium sensu strico 1	D28 feces	Wet/dry	7.50
Clostridium sensu strico 1	D28 feces	Dry	7.16
Lactobacillus	D28 feces	Dry	6.87
Clostridium sensu strico 1	D28 feces	Liquid	6.67
Streptococcus	D28 feces	Dry	6.64
Prevotellaceae NK3B31 group	D28 feces	Dry	6.16
Prevotellaceae NK3B31 group	D28 feces	Liquid	6.15
Megasphaera	D28 feces	Wet/dry	5.11
Prevotellaceae NK3B31 group	D28 feces	Wet/dry	4.76

Lactobacillus	D28 feces	Wet/dry	4.60
Megasphaera	D28 feces	Liquid	4.26
Muribaculaceae	D28 feces	Liquid	4.15
Muribaculaceae	D28 feces	Dry	3.94
Muribaculaceae	D28 feces	Wet/dry	3.75
Megasphaera	D28 feces	Dry	3.73
Rikenellaceae RC9 gut group	D28 feces	Dry	3.54
Streptococcus	D28 feces	Liquid	3.50
Rikenellaceae RC9 gut group	D28 feces	Liquid	3.27
Rikenellaceae RC9 gut group	D28 feces	Wet/dry	3.24
Terrisporobacter	D28 feces	Wet/dry	2.47
Alloprevotella	D28 feces	Dry	2.44
Subdoligranulum	D28 feces	Liquid	2.42
Alloprevotella	D28 feces	Wet/dry	2.42
Alloprevotella	D28 feces	Liquid	2.38
Subdoligranulum	D28 feces	Dry	2.11
Anaerovibrio	D28 feces	Liquid	2.09
Treponema	D28 feces	Liquid	2.02
Subdoligranulum	D28 feces	Wet/dry	2.01
Terrisporobacter	D28 feces	Liquid	1.99
UCG-005	D28 feces	Liquid	1.96
Treponema	D28 feces	Dry	1.89
Blautia	D28 feces	Dry	1.85
Phascolarctobacterium	D28 feces	Liquid	1.82
Terrisporobacter	D28 feces	Dry	1.81
Blautia	D28 feces	Liquid	1.78
Blautia	D28 feces	Wet/dry	1.70
Anaerovibrio	D28 feces	Wet/dry	1.70
UCG-005	D28 feces	Dry	1.68
Phascolarctobacterium	D28 feces	Dry	1.67
Anaerovibrio	D28 feces	Dry	1.63
Christensenellaceae R-7 group	D28 feces	Dry	1.57
Phascolarctobacterium	D28 feces	Wet/dry	1.57
Christensenellaceae R-7 group	D28 feces	Liquid	1.55
Prevotellaceae UCG-003	D28 feces	Liquid	1.53
Ruminococcus	D28 feces	Dry	1.51
UCG-005	D28 feces	Wet/dry	1.45
Prevotellaceae UCG-003	D28 feces	Dry	1.42
Roseburia	D28 feces	Liquid	1.41
Faecalibacterium	D28 feces	Wet/dry	1.39
Roseburia	D28 feces	Wet/dry	1.38
Ruminococcus	D28 feces	Wet/dry	1.37
Ruminococcus	D28 feces	Liquid	1.27
Faecalibacterium	D28 feces	Dry	1.23

Acidaminococcus	D28 feces	Wet/dry	1.22
Roseburia	D28 feces	Dry	1.21
Christensenellaceae R-7 group	D28 feces	Wet/dry	1.19
Prevotellaceae UCG-003	D28 feces	Wet/dry	1.17
Dialister	D28 feces	Wet/dry	1.15
Treponema	D28 feces	Wet/dry	1.14
Faecalibacterium	D28 feces	Liquid	1.03
Prevotella	D63 feces	Liquid	17.00
Prevotella	D63 feces	Wet/dry	9.08
Prevotella	D63 feces	Dry	8.88
Prevotellaceae NK3B31 group	D63 feces	Dry	8.38
Prevotellaceae NK3B31 group	D63 feces	Liquid	7.39
Rikenellaceae RC9 gut group	D63 feces	Wet/dry	7.18
Clostridium sensu strico 1	D63 feces	Wet/dry	7.11
Clostridium sensu strico 1	D63 feces	Dry	7.06
Treponema	D63 feces	Wet/dry	6.07
Rikenellaceae RC9 gut group	D63 feces	Dry	5.83
Muribaculaceae	D63 feces	Dry	5.69
Christensenellaceae R-7 group	D63 feces	Wet/dry	5.11
Treponema	D63 feces	Dry	5.05
Christensenellaceae R-7 group	D63 feces	Dry	4.99
Prevotellaceae NK3B31 group	D63 feces	Wet/dry	4.90
Muribaculaceae	D63 feces	Wet/dry	4.89
Rikenellaceae RC9 gut group	D63 feces	Liquid	4.79
Muribaculaceae	D63 feces	Liquid	4.60
Clostridium sensu strico 1	D63 feces	Liquid	3.86
Streptococcus	D63 feces	Liquid	3.80
Treponema	D63 feces	Liquid	3.77
Lactobacillus	D63 feces	Liquid	3.59
Streptococcus	D63 feces	Dry	3.36
Streptococcus	D63 feces	Wet/dry	2.97
Christensenellaceae R-7 group	D63 feces	Liquid	2.87
Prevotellaceae UCG-001	D63 feces	Wet/dry	2.72
Alloprevotella	D63 feces	Dry	2.58
Megasphaera	D63 feces	Liquid	2.54
Alloprevotella	D63 feces	Liquid	2.44
Alloprevotella	D63 feces	Wet/dry	2.39
UCG-010	D63 feces	Wet/dry	2.31
Prevotellaceae UCG-003	D63 feces	Wet/dry	2.20
Prevotellaceae UCG-003	D63 feces	Dry	2.11
Bacteroidales RF16 group	D63 feces	Dry	2.05
UCG-010	D63 feces	Dry	1.96
Terrisporobacter	D63 feces	Wet/dry	1.94
UCG-005	D63 feces	Wet/dry	1.94

Terrisporobacter	D63 feces	Dry	1.84
UCG-005	D63 feces	Dry	1.79
Phascolarctobacterium	D63 feces	Liquid	1.77
Prevotellaceae UCG-003	D63 feces	Liquid	1.73
Parabacteroides	D63 feces	Wet/dry	1.65
Terrisporobacter	D63 feces	Liquid	1.65
UCG-005	D63 feces	Liquid	1.61
Anaerovibrio	D63 feces	Liquid	1.58
Phascolarctobacterium	D63 feces	Dry	1.56
UCG-002	D63 feces	Wet/dry	1.56
UCG-010	D63 feces	Liquid	1.54
Clostridia vadinBB60 group	D63 feces	Wet/dry	1.54
Parabacteroides	D63 feces	Dry	1.53
Lactobacillus	D63 feces	Dry	1.47
UCG-002	D63 feces	Dry	1.46
Clostridia vadinBB60 group	D63 feces	Dry	1.46
dgA-11 gut group	D63 feces	Wet/dry	1.41
Phascolarctobacterium	D63 feces	Wet/dry	1.39
Ruminococcus	D63 feces	Wet/dry	1.21
Ruminococcus	D63 feces	Liquid	1.20
WCHB1-41	D63 feces	Wet/dry	1.18
Prevotellaceae UCG-001	D63 feces	Dry	1.17
Prevotellaceae UCG-001	D63 feces	Liquid	1.15
UCG-002	D63 feces	Liquid	1.13
Fibrobacter	D63 feces	Wet/dry	1.12
Anaerovibrio	D63 feces	Dry	1.12
p-2534-18B5 gut group	D63 feces	Liquid	1.11
Eubacterium coprostanoligenes group	D63 feces	Wet/dry	1.11
Ruminococcus	D63 feces	Dry	1.10
NK4A214 group	D63 feces	Wet/dry	1.08
Lactobacillus	D63 feces	Wet/dry	1.05
p-2534-18B5 gut group	D63 feces	Wet/dry	1.04
WCHB1-41	D63 feces	Dry	1.03
Subdoligranulum	D63 feces	Liquid	1.02
Megasphaera	D63 feces	Dry	1.00
Eubacterium coprostanoligenes group	D63 feces	Dry	1.00
Clostridia vadinBB60 group	D63 feces	Liquid	1.00

Supplementary Table S5: Bacterial genera differentially abundant between treatment groups in the intestinal digesta and feces of grow-finisher pigs fed the experimental diets, following pairwise comparisons in DESeq2.

Treatment comparison[1]	Sample type	Genus	Base mean	Log2-fold-change	lfcSE[2]	Stat	*P*-value[3]	Enriched group
DM - DP	Ileal digesta	*Mitsuokella*	7245.05	8.56	1.50	5.71	8.61E-08	DM
DM - DP	Ileal digesta	*Megasphaera*	8018.46	7.98	1.50	5.32	6.92E-07	DM
DM - DP	Ileal digesta	*Prevotella*	4611.63	6.02	1.42	4.26	7.53E-05	DM
DM - LP	Ileal digesta	*Lactobacillus*	18643.69	4.61	1.09	4.23	7.33E-05	DM
DM - LP	Ileal digesta	*Mitsuokella*	7245.05	9.85	1.41	6.97	1.80E-11	DM
DM - LP	Ileal digesta	*Megasphaera*	8018.46	9.45	1.41	6.68	1.22E-10	DM
DM - LP	Ileal digesta	*Prevotella*	4611.63	6.84	1.33	5.13	1.26E-06	DM
DM - WDM	Ileal digesta	*Mitsuokella*	7245.05	3.70	1.45	2.55	3.62E-02	DM
DM - WDM	Ileal digesta	*Megasphaera*	8018.46	4.91	1.45	3.38	3.38E-03	DM
DM - WDM	Ileal digesta	*Prevotella*	4611.63	6.41	1.37	4.68	1.94E-05	DM
DM - WDP	Ileal digesta	*Lactobacillus*	18643.69	3.09	1.20	2.56	2.22E-02	WDP
DM - WDP	Ileal digesta	*Mitsuokella*	7245.05	11.94	1.58	7.56	3.82E-13	DM
DM - WDP	Ileal digesta	*Megasphaera*	8018.46	10.36	1.57	6.61	2.24E-10	DM
DM - WDP	Ileal digesta	*Prevotella*	4611.63	9.08	1.48	6.14	4.22E-09	DM
DP - LM	Ileal digesta	*Escherichia-Shigella*	4857.56	2.17	0.70	3.09	5.83E-03	DP
DP - LM	Ileal digesta	*Mitsuokella*	7245.05	-6.83	1.45	-4.71	9.74E-06	LM
DP - LM	Ileal digesta	*Megasphaera*	8018.46	-7.07	1.45	-4.87	5.17E-06	LM
DP - LM	Ileal digesta	*Prevotella*	4611.63	-4.85	1.37	-3.54	1.35E-03	LM
DP - LP	Ileal digesta	*Lactobacillus*	18643.69	4.95	1.09	4.54	4.45E-05	DP
DP - LP	Ileal digesta	*Escherichia-Shigella*	4857.56	2.50	0.68	3.66	1.70E-03	DP
DP - WDM	Ileal digesta	*Lactobacillus*	18643.69	2.91	1.12	2.60	2.45E-02	DP
DP - WDM	Ileal digesta	*Escherichia-Shigella*	4857.56	2.16	0.70	3.08	6.93E-03	DP
DP - WDM	Ileal digesta	*Mitsuokella*	7245.05	-4.87	1.45	-3.35	2.87E-03	WDM
DP - WDP	Ileal digesta	*Lactobacillus*	18643.69	3.42	1.20	2.84	3.02E-02	DP

LM - LP	Ileal digesta	*Lactobacillus*	18643.69	4.97	1.05	4.72	1.38E-05	LM
LM - LP	Ileal digesta	*Mitsuokella*	7245.05	8.12	1.36	5.96	2.33E-08	LM
LM - LP	Ileal digesta	*Megasphaera*	8018.46	8.54	1.36	6.27	4.32E-09	LM
LM - LP	Ileal digesta	*Prevotella*	4611.63	5.66	1.29	4.40	5.54E-05	LM
LM - WDM	Ileal digesta	*Lactobacillus*	18643.69	2.93	1.08	2.70	3.04E-02	LM
LM - WDM	Ileal digesta	*Megasphaera*	8018.46	4.00	1.40	2.86	2.24E-02	LM
LM - WDM	Ileal digesta	*Prevotella*	4611.63	5.24	1.32	3.96	5.12E-04	LM
LM - WDP	Ileal digesta	*Lactobacillus*	18643.69	3.44	1.17	2.94	9.08E-03	LM
LM - WDP	Ileal digesta	*Mitsuokella*	7245.05	10.21	1.54	6.65	2.70E-10	LM
LM - WDP	Ileal digesta	*Megasphaera*	8018.46	9.46	1.52	6.21	4.03E-09	LM
LM - WDP	Ileal digesta	*Prevotella*	4611.63	7.91	1.44	5.51	2.42E-07	LM
LP - WDM	Ileal digesta	*Mitsuokella*	7245.05	-6.16	1.36	-4.52	4.85E-05	WDM
LP - WDM	Ileal digesta	*Megasphaera*	8018.46	-4.54	1.36	-3.33	5.08E-03	WDM
WDM - WDP	Ileal digesta	*Mitsuokella*	7245.05	8.25	1.54	5.37	6.09E-07	WDM
WDM - WDP	Ileal digesta	*Megasphaera*	8018.46	5.46	1.52	3.58	1.76E-03	WDM
DM - DP	Caecal digesta	*Clostridium sensu strico 1*	10794.55	0.80	0.23	3.53	7.14E-03	DM
DM - LM	Caecal digesta	*Clostridium sensu strico 1*	10794.55	0.99	0.22	4.51	2.24E-04	DM
DM - LM	Caecal digesta	*Lactobacillus*	3890.41	-1.86	0.66	-2.82	4.38E-02	LM
DM - LP	Caecal digesta	*Clostridium sensu strico 1*	10794.55	0.75	0.21	3.51	1.02E-02	DM
DM - LP	Caecal digesta	*Mitsuokella*	941.82	2.19	0.69	3.19	2.41E-02	DM
DP - LM	Caecal digesta	*Streptococcus*	3813.57	2.07	0.55	3.79	2.27E-03	DP
DP - LM	Caecal digesta	*Lactobacillus*	3890.41	-2.26	0.66	-3.41	8.80E-03	LM
DP - LM	Caecal digesta	*Escherichia-Shigella*	766.55	3.46	0.78	4.42	1.64E-04	DP
LM - LP	Caecal digesta	*Lactobacillus*	3890.41	3.48	0.62	5.61	5.63E-07	LM
LM - LP	Caecal digesta	*Escherichia-Shigella*	766.55	-2.49	0.73	-3.39	1.04E-02	LP
LM - WDM	Caecal digesta	*Clostridium sensu strico 1*	10794.55	-0.73	0.21	-3.43	1.04E-02	WDM
LM - WDM	Caecal digesta	*Streptococcus*	3813.57	-1.64	0.53	-3.11	2.49E-02	WDM
LM - WDM	Caecal digesta	*Lactobacillus*	3890.41	3.23	0.64	5.05	1.98E-05	LM

LM - WDM	Caecal digesta	*Escherichia-Shigella*	766.55	-2.68	0.75	-3.55	7.35E-03	WDM
LM - WDP	Caecal digesta	*Streptococcus*	3813.57	-2.18	0.57	-3.83	2.21E-03	WDP
LM - WDP	Caecal digesta	*Escherichia-Shigella*	766.55	-2.55	0.82	-3.12	2.44E-02	WDP
DM - DP	D28 feces	*Streptococcus*	3065.63	-1.17	0.35	-3.33	1.26E-02	DP
DM - LM	D28 feces	*Streptococcus*	3065.63	1.97	0.35	5.60	3.15E-06	DM
DM - WDP	D28 feces	*Prevotella*	7562.87	-1.03	0.28	-3.66	1.90E-03	WDP
DM - WDP	D28 feces	*Lactobacillus*	3588.67	1.55	0.44	3.54	2.83E-03	DM
DM - WDP	D28 feces	*Streptococcus*	3065.63	-1.57	0.35	-4.46	1.07E-04	WDP
DM - WDP	D28 feces	*Megasphaera*	2201.20	-1.69	0.43	-3.91	7.68E-04	WDP
DM - WDP	D28 feces	*Mitsuokella*	229.45	-2.55	0.77	-3.32	5.44E-03	WDP
DP - LM	D28 feces	*Lactobacillus*	3588.67	-1.76	0.44	-4.02	1.68E-03	LM
DP - LM	D28 feces	*Streptococcus*	3065.63	3.15	0.35	8.92	3.40E-17	DP
DP - LP	D28 feces	*Escherichia-Shigella*	24.28	4.45	1.18	3.77	1.15E-02	DP
DP - WDM	D28 feces	*Prevotella*	7562.87	0.82	0.28	2.91	4.26E-02	DP
DP - WDM	D28 feces	*Clostridium sensu strico 1*	3319.02	-0.81	0.29	-2.83	4.82E-02	WDM
LM - LP	D28 feces	*Lactobacillus*	3588.67	1.33	0.44	3.03	3.09E-02	LM
LM - LP	D28 feces	*Streptococcus*	3065.63	-2.45	0.35	-6.95	2.16E-10	LP
LM - LP	D28 feces	*Escherichia-Shigella*	24.28	3.67	1.18	3.11	2.86E-02	LM
LM - WDM	D28 feces	*Clostridium sensu strico 1*	3319.02	-0.92	0.29	-3.21	3.79E-02	WDM
LM - WDM	D28 feces	*Streptococcus*	3065.63	-2.45	0.35	-6.95	5.15E-10	WDM
LM - WDP	D28 feces	*Prevotella*	7562.87	-0.97	0.28	-3.44	4.05E-03	WDP
LM - WDP	D28 feces	*Lactobacillus*	3588.67	2.51	0.44	5.73	3.35E-07	LM
LM - WDP	D28 feces	*Streptococcus*	3065.63	-3.54	0.35	-10.05	6.32E-22	WDP
LP - WDM	D28 feces	*Prevotella*	7562.87	0.87	0.28	3.08	2.96E-02	LP
LP - WDP	D28 feces	*Streptococcus*	3065.63	-1.09	0.35	-3.11	2.07E-02	WDP
LP - WDP	D28 feces	*Megasphaera*	2201.20	-1.22	0.43	-2.83	4.22E-02	WDP
LP - WDP	D28 feces	*Mitsuokella*	229.45	-2.55	0.77	-3.32	1.59E-02	WDP
LP - WDP	D28 feces	*Escherichia-Shigella*	24.28	-3.91	1.18	-3.32	1.59E-02	WDP

WDM - WDP	D28 feces	*Prevotella*	7562.87	-1.25	0.28	-4.42	1.42E-04	WDP
WDM - WDP	D28 feces	*Lactobacillus*	3588.67	1.28	0.44	2.92	1.70E-02	WDM
WDM - WDP	D28 feces	*Clostridium sensu strico 1*	3319.02	0.86	0.29	3.01	1.39E-02	WDM
WDM - WDP	D28 feces	*Streptococcus*	3065.63	-1.09	0.35	-3.10	1.10E-02	WDP
WDM - WDP	D28 feces	*Megasphaera*	2201.20	-1.30	0.43	-3.02	1.39E-02	WDP
WDM - WDP	D28 feces	*Mitsuokella*	229.45	-2.90	0.77	-3.77	1.29E-03	WDP
DM - DP	D63 feces	*Megasphaera*	737.78	-3.70	0.86	-4.28	6.34E-04	DP
DM - LM	D63 feces	*Prevotella*	4665.16	-2.08	0.47	-4.40	2.33E-04	LM
DM - LM	D63 feces	*Lactobacillus*	906.76	-3.09	0.67	-4.62	1.17E-04	LM
DM - LM	D63 feces	*Megasphaera*	737.78	-5.23	0.86	-6.06	1.07E-07	LM
DM - LM	D63 feces	*Mitsuokella*	73.05	-7.05	1.62	-4.35	2.51E-04	LM
DM - LP	D63 feces	*Prevotella*	4665.16	-1.89	0.47	-4.01	1.33E-03	LP
DM - LP	D63 feces	*Clostridium sensu strico 1*	2011.12	1.12	0.30	3.70	3.68E-03	DM
DM - LP	D63 feces	*Megasphaera*	737.78	-2.55	0.86	-2.96	2.49E-02	LP
DM - LP	D63 feces	*Mitsuokella*	73.05	-4.94	1.62	-3.04	2.15E-02	LP
DM - WDP	D63 feces	*Prevotella*	4665.16	-1.57	0.47	-3.32	1.51E-02	WDP
DM - WDP	D63 feces	*Megasphaera*	737.78	-2.89	0.86	-3.35	1.51E-02	WDP
DM - WDP	D63 feces	*Mitsuokella*	73.05	-4.56	1.62	-2.81	4.25E-02	WDP
DP - LM	D63 feces	*Lactobacillus*	906.76	-2.71	0.67	-4.05	1.90E-03	LM
DP - WDM	D63 feces	*Prevotella*	4665.16	2.05	0.47	4.35	4.06E-04	DP
DP - WDM	D63 feces	*Clostridium sensu strico 1*	2011.12	-0.80	0.30	-2.65	4.93E-02	WDM
DP - WDM	D63 feces	*Streptococcus*	1441.57	2.22	0.72	3.08	2.22E-02	DP
DP - WDM	D63 feces	*Megasphaera*	737.78	4.48	0.87	5.18	1.13E-05	DP
DP - WDM	D63 feces	*Escherichia-Shigella*	42.59	2.29	0.84	2.75	4.02E-02	DP
LM - LP	D63 feces	*Lactobacillus*	906.76	3.36	0.67	5.03	2.95E-05	LM
LM - LP	D63 feces	*Megasphaera*	737.78	2.68	0.86	3.10	3.55E-02	LM
LM - WDM	D63 feces	*Prevotella*	4665.16	2.84	0.47	6.03	6.40E-08	LM
LM - WDM	D63 feces	*Clostridium sensu strico 1*	2011.12	-0.77	0.30	-2.53	3.83E-02	WDM

LM - WDM	D63 feces	*Streptococcus*	1441.57	2.53	0.72	3.51	2.53E-03	LM
LM - WDM	D63 feces	*Lactobacillus*	906.76	3.18	0.67	4.75	3.78E-05	LM
LM - WDM	D63 feces	*Megasphaera*	737.78	6.02	0.87	6.95	2.78E-10	LM
LM - WDM	D63 feces	*Mitsuokella*	73.05	5.10	1.60	3.20	6.39E-03	LM
LM - WDM	D63 feces	*Escherichia-Shigella*	42.59	2.01	0.84	2.40	4.93E-02	LM
LM - WDP	D63 feces	*Lactobacillus*	906.76	3.66	0.67	5.47	3.37E-06	LM
LP - WDM	D63 feces	*Prevotella*	4665.16	2.66	0.47	5.63	9.05E-07	LP
LP - WDM	D63 feces	*Clostridium sensu strico 1*	2011.12	-1.24	0.30	-4.10	5.68E-04	WDM
LP - WDM	D63 feces	*Megasphaera*	737.78	3.34	0.87	3.86	1.25E-03	LP
WDM - WDP	D63 feces	*Prevotella*	4665.16	-2.33	0.47	-4.95	2.82E-05	WDP
WDM - WDP	D63 feces	*Clostridium sensu strico 1*	2011.12	0.85	0.30	2.81	3.09E-02	WDM
WDM - WDP	D63 feces	*Streptococcus*	1441.57	-2.03	0.72	-2.81	3.09E-02	WDP
WDM - WDP	D63 feces	*Megasphaera*	737.78	-3.68	0.87	-4.25	4.01E-04	WDP

[1] Treatment groups are abbreviated as follows: DM = Dry meal-fed pigs, DP = Dry pellet-fed pigs, LM = Liquid meal-fed pigs, LP = Liquid pellet-fed pigs, WDM = Wet/dry meal-fed pigs, WDP = Wet/dry pellet-fed pigs.

[2] lfcSE: Standard error of the $\log_2$ fold-change.

[3] Benjamini-Hochberg adjusted *P*-value.
